# Capturing expert uncertainty in spatial cumulative impact assessments

**DOI:** 10.1038/s41598-018-19354-6

**Published:** 2018-01-23

**Authors:** Alice R. Jones, Zoë A. Doubleday, Thomas A. A. Prowse, Kathryn H. Wiltshire, Marty R. Deveney, Tim Ward, Sally L. Scrivens, Phillip Cassey, Laura G. O’Connell, Bronwyn M. Gillanders

**Affiliations:** 1 The University of Adelaide, School of Biological Sciences and Environment Institute, Adelaide, SA 5005 Australia; 2 South Australian Research and Development Institute, Aquatic Sciences, West Beach, SA 5024 Australia; 3 Department of Geological Sciences and Geological Engineering, Queen’s University, Kingston, K7L 3N6 Ontario Canada; 4 Present Address: The University of Adelaide, School of Mathematical Sciences, Adelaide, SA 5005 Australia; 5 Present Address: Geology, Southern Illinois University, Carbondale, 62901 Illinois USA

## Abstract

Understanding the spatial distribution of human impacts on marine environments is necessary for maintaining healthy ecosystems and supporting ‘blue economies’. Realistic assessments of impact must consider the cumulative impacts of multiple, coincident threats and the differing vulnerabilities of ecosystems to these threats. Expert knowledge is often used to assess impact in marine ecosystems because empirical data are lacking; however, this introduces uncertainty into the results. As part of a spatial cumulative impact assessment for Spencer Gulf, South Australia, we asked experts to estimate score ranges (best-case, most-likely and worst-case), which accounted for their uncertainty about the effect of 32 threats on eight ecosystems. Expert scores were combined with data on the spatial pattern and intensity of threats to generate cumulative impact maps based on each of the three scoring scenarios, as well as simulations and maps of uncertainty. We compared our method, which explicitly accounts for the experts’ knowledge-based uncertainty, with other approaches and found that it provides smaller uncertainty bounds, leading to more constrained assessment results. Collecting these additional data on experts’ knowledge-based uncertainty provides transparency and simplifies interpretation of the outputs from spatial cumulative impact assessments, facilitating their application for sustainable resource management and conservation.

## Introduction

Over 97% of the world’s oceans are exposed to multiple concurrent threats from human activities resulting in cumulative impacts^1^, with the severity of these cumulative impacts increasing in recent years^2,3^. Shelf seas are particularly at-risk because of their vulnerability to both terrestrial and marine threats^1,4,5^, which generally fall into four categories: pollution (including climate change), over-extraction, physical degradation and invasive species.

Spatial cumulative impact assessment is increasingly being used as a tool for evaluating the effect of multiple anthropogenic threats on marine ecosystems^6^. The approach accounts for both the vulnerability of marine ecosystems to different threats^7^ and the spatial exposure and intensity of each threat throughout a defined study area^1^. As such, this type of assessment can support integrated management approaches that monitor and counter multiple threats, rather than single threats in isolation^8–10^. The utility of these assessments is affected by the spatial scale at which ecosystems and threats are mapped and the relevance of this scale to management, which is usually locally or regionally targeted^11,12^.

Since empirical data are often scarce^13^, cumulative impact assessments typically rely on expert knowledge to score and rank the effect that each threat may have on each ecosystem^6,14^. Although methodologies exist for incorporating expert knowledge into spatial cumulative impact assessments, these do not routinely account for the ‘knowledge-based uncertainty’ (see glossary in Table 1) associated with expert-elicited data^1,7^. Knowledge-based uncertainty has the potential to affect the reliability of the assessment results^15,16^ and their application to management^13,14^.

**Table 1:** Glossary of terms used throughout this paper.

GLOSSARY	
Term	Definition/explanation
*Ecosystem*	The benthic (n = 7) or pelagic ecosystem present in each grid cell (250 × 250 m) within the Spencer Gulf study area. The ecosystem type attributed to each grid cell was determined by the dominant ecosystem in the cell (based on percentage area covered).
*Threat*	A human activity or climatic perturbation, which is considered to be a threat to one or more of the eight ecosystems within the study area based on published research or expert knowledge^22^.
*Effect score*	The expert-elicited score for the effect of each threat on each ecosystem (collected through N = 81 online surveys – see^22^). Experts provided three effect scores for each ecosystem and threat pair: ‘worst-case’ scenario (higher scores), ‘most-likely’ scenario (middle scores) and ‘best-case’ scenario (lower scores). The range between the ‘best-case’ and ‘worst case’ effect scores represents the experts’ uncertainty bounds for the most-likely effect of a threat on an ecosystem.
*Spatial exposure*	A binary value (0 or 1) representing the absence (0) or presence (1) of a threat at a given grid cell (250 × 250 m). These spatial layers indicate the area within which a threat occurs *at any intensity*. The spatial exposure scores can be summed at any location to provide a count of the number of threats present there.
*Spatial intensity score*	A continuous score, between 0 and 1, which represents the relative intensity of a threat at any grid cell within the study area. The cell with the highest spatial intensity score (1) for each threat is the location where this threat occurs at the greatest intensity. For example, for lobster pot fishing, this would be the location with the highest average number of pots dropped. In locations where a threat does not occur, the spatial exposure *and* spatial intensity scores are both 0.
*Cumulative impact score*	A score representing the additive effect of all threats occurring at each location. The cumulative impact score is calculated using the experts’ effect scores and the location-specific spatial intensity scores (see Eq. 1). A cumulative impact score for each location was calculated for each of the three effect score scenarios (‘best-case’, ‘most-likely’ and ‘worst-case’).
*Knowledge-based uncertainty*	The self-assessed level of uncertainty associated with effect scores provided through expert elicitation surveys. Experts were asked to give three effect scores relating to best-case, most-likely and worst-case scenarios (see ‘*effect score*’ definition above and Doubleday *et al*.^22^). This allowed each expert to provide what they considered to be a plausible range of effect scores, which expressed their level of uncertainty in the effect of each threat on each ecosystem. We have termed this ‘knowledge-based uncertainty’ as it represents uncertainty introduced into the cumulative impact assessment because of limited or imperfect expert knowledge or scientific understanding^15^ (epistemic uncertainty). However this uncertainty may be confounded with natural variation (aleatory uncertainty) and linguistic uncertainty^41^, which cannot easily be separated from epistemic uncertainty^20^.

Sensitivity testing is commonly performed to check the overall influence of each threat on the final results of spatial cumulative impact assessments (for example Korpinen *et al*.^17^), but quantification of the influence of knowledge-based uncertainty has not been adopted into the commonly-used cumulative impact assessment methodologies^6,18^. Understanding uncertainty in expert-elicited data is crucial to interpreting the results of cumulative impact assessments and can also be used to highlight regions or ecosystems where uncertainty is particularly high and further data are needed. Stock and Micheli^19^ and Gissi *et al*.^18^ both recently investigated the impact of various types of uncertainty on marine spatial cumulative impact assessment outcomes. Stock and Micheli^19^ simulated error (uncertainty) around expert-elicited scores after the expert elicitation process had been undertaken. Whilst this is an important step forward for cumulative impact assessment methods, it sets the boundaries of expert uncertainty *a priori*, rather than asking experts to quantify their uncertainty themselves (i.e. to ‘self-assess’ their level of uncertainty). Conversely, Gissi *et al*.^18^ collected various uncertainty information as a central part of the expert elicitation process. By recognising that knowledge-based uncertainty exists, and asking experts to attempt to quantify it in a robust way^20,21^, researchers can generate data that explicitly accounts for this source of uncertainty in assessment outputs^15^.

We undertook a spatial cumulative impact assessment for Spencer Gulf, South Australia, based on the method developed by Halpern *et al*.^1^. We used a survey to elicit expert knowledge for our assessment. The survey allowed experts to self-assess uncertainty by supplying a range of plausible values for the effect of a threat on an ecosystem^22^. We demonstrate how these additional data on knowledge-based uncertainty can be used to improve the robustness of cumulative impact assessments and the ease of their interpretation.

## Methods

### Study Area

Spencer Gulf is Australia’s largest estuary, covering approximately 30,000 km^2^ ^23^. It is an inverse estuary, where salinity increases with distance from the open sea because evaporation exceeds precipitation in this semi-arid region^24^. The marine ecosystems of Spencer Gulf are diverse and contain a high proportion of endemic species^25^. Gulf waters are widely used for recreation and tourism and contain eight marine parks (including 23 no-take sanctuary zones). Spencer Gulf is also extremely important to the region’s economy^26^, being responsible for producing over half of South Australia’s seafood^27,28^ and providing a key shipping and export gateway for the energy, mining and agriculture industries^29^.

### Mapping marine ecosystems

We identified ten broad-scale benthic (seafloor) ecosystems within Spencer Gulf^22,26^, however three of these (sponge gardens, rhodolith beds and native shellfish beds) could not be included in the analysis, due to limited spatial data. The few data available indicate that these ecosystems are relatively small in extent, however they may be particularly vulnerable to human activities^30–33^. We collated all available spatial data on the remaining seven benthic ecosystems (Supplementary materials section S1.1 & Supplementary Table S1) to produce the first, full-coverage, broad-scale benthic ecosystem map for Spencer Gulf (Supplementary Figure S1). We also generated a map indicating the level of confidence in the ecosystem classification, which is based on the type and resolution of the available data in each area of the Gulf and how recently data were collected (Supplementary Figure S1). The benthic ecosystem map was rasterised to a grid of 250 × 250 m, with each cell assigned the dominant ecosystem type. We separately mapped a pelagic (water column) ecosystem, delineated by the limit of subtidal waters (Supplementary Figure S2). We assessed cumulative impacts separately for the pelagic ecosystem because there was considerable overlap of the benthic (seafloor) and pelagic (water column) ecosystems (Supplementary Figures S1 & S2).

### Mapping threats

Thirty-two threats were included in the spatial cumulative impact assessment. These were selected through expert consultation and a literature review^22^. Threats included regional-scale and relevant global-scale human activities and processes for which we could access, or generate, Gulf-wide spatial data (see online Supplementary material S1.2). The suite of threats included activities related to land use and development, recreation, commercial fishing, shipping, pollution, invasive species and climate change (for a full list and data sources see Supplementary Table S2). We did not transform the threat data layers, so as to retain realistic threat intensity patterns and extreme values, which may be relevant in an analysis of how ecosystems are impacted by threat activities^6^. When data were available, we mapped threats directly (e.g. nutrient inputs), but in some cases proxies were used (e.g. an impact kernel around port developments). For most threats, relative spatial intensity (see glossary in Table 1) was mapped, but in some cases only the spatial exposure (presence or absence, see glossary in Table 1) of a threat at each location was mapped (due to poor data quality or resolution; Supplementary Table S2; Supplementary Figure S3). We transformed the spatial layers to a common projection (unit of measurement = m) and then rasterised them to a 250-m grid resolution by up- or down-scaling as necessary. All data processing and visualisation was conducted in the R software environment for statistical and graphical computing (version 3.3.1)^34^ using packages ‘sp’^35^, ‘raster’^36^, ‘rgdal’^37^ and ‘rasterVis’^38^.

### Calculating cumulative impact

We used expert knowledge collected through online surveys (N = 81) to generate scores for the effect of each threat on each ecosystem (${\mu }_{{ij}}$); these are referred to as ‘effect scores’ (see glossary, Table 1). For the purposes of this assessment, experts were defined as people with expertise in relevant ecosystems and an understanding of South Australia’s gulf environments. Experts had a range of qualifications and experience, and were selected from various fields including academia (researchers, academics and postgraduate students), state government and environmental consultancy. However, we recognise that a broader definition of ‘experts’ would have allowed for greater inclusion of, for example, fishers, interested citizens, NGOs and industry representatives^39^.

To capture data on knowledge-based uncertainty, experts were asked to give a range of effect scores for each ecosystem-threat combination, relating to different scoring scenarios: ‘best-case’ (generally the lowest scores), ‘most-likely’ (best estimate) and ‘worst-case’ (generally the highest scores). The experts were provided with a survey reference sheet that explained the meaning of these scenarios in terms of their level of uncertainty about the effect of a threat on an ecosystem (available in the Supplementary material for Doubleday *et al*.^22^). The three scenario scores could be very different if experts were highly uncertain of the effect, or very similar (even the same) if the expert was highly certain of the effect.

We encouraged the surveyed experts to contact the research team (via phone or email) with any queries, or requests for clarification regarding the questions in the online survey. Even so, there is potential for language-based (linguistic) uncertainty to have been introduced into the results, because of differences in interpretation of potentially vague, ambiguous, context-dependent, generalised or indeterminate terms and/or counterfactual statements in the survey questions^40^. This linguistic uncertainty can lead to further uncertainty and variance in the expert-elicited effect scores, potentially leading to an artificial narrowing of the certainty bounds around the most-likely effect scores^41^. The full methodology of the expert elicitation, along with copies of the online surveys and additional information provided to the experts is available in Doubleday *et al*.^22^.

**Figure 1:**
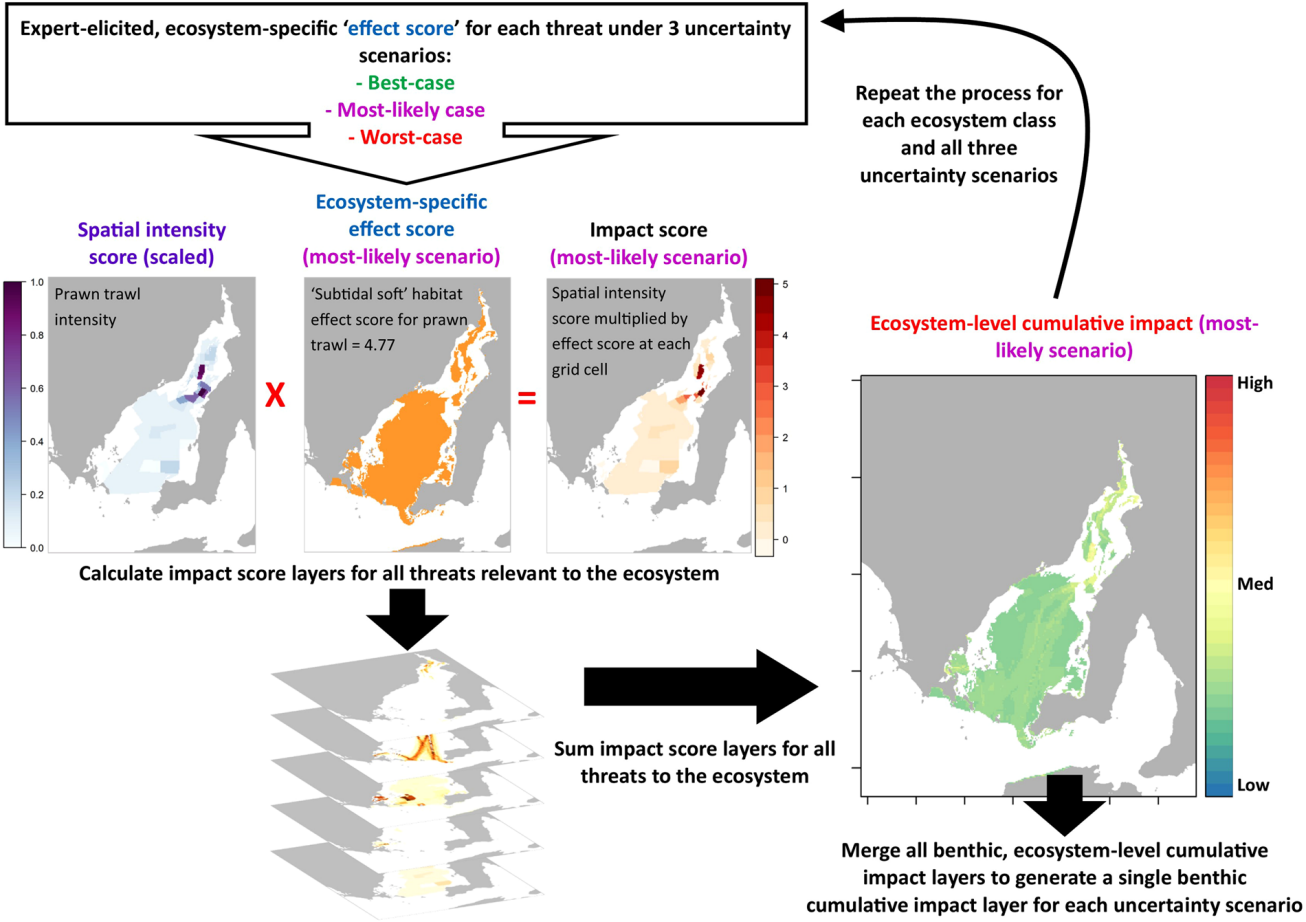
Schematic of framework for calculating cumulative impact and accounting for ‘knowledge-based uncertainty’. Experts were asked to give ‘effect scores’ (between 0–8) for each combination of threat (in this example it is prawn trawl fishing) and ecosystem (here subtidal soft sediment), and for three uncertainty scenarios:’best-case’, ‘most-likely’ and ‘worst-case’. These effect scores were then used in a calculation of impact that accounts for the spatial intensity of each threat and the locations of overlap between a threat and an ecosystem. This process was repeated for each threat that occurs to each ecosystem and the resulting impact layers were summed to generate ecosystem-specific cumulative impact maps. This entire process was carried out for all eight ecosystems, and was repeated three times, once for each uncertainty scenario, to account for the experts’ ‘knowledge-based uncertainty’ around the cumulative impact scores. Maps were produced using R statistical software (version 3.3.1; https://www.r-project.org) and the packages raster^36^, rgdal^37^, sp^35^ and rasterVis^38^.

We followed the general framework developed by Halpern *et al*. and described in detail in their papers^1,7^. Our adapted version of their method calculates the cumulative impact score for the eight ecosystems separately (Eq. 1).

1 $$C{I}^{s}=\sum _{(i=1)}^{n}{D}_{i}\times {\mu }_{i,e}$$

where *CI*^*s*^ is the cumulative impact at each grid cell based on effect score scenario *s* (either, ‘best-case’, ‘most-likely’ or ‘worst-case’). *D*_*i*_ is the spatial intensity of threat *i* (scaled between 0 and 1, with 1 representing the highest value for the threat) and *µ*_*i*,*e*_ is the average, expert-elicited effect score for threat *i* on ecosystem *e* (score range = 0–8) calculated from all experts’ scores for the scenario, threat and ecosystem in question (Fig. 1). There were N = 32 threats in our assessment, but not all ecosystems were exposed to all threats (Supplementary Figures S1 & S4). Eq. 1 results in a value of 0 for a threat-ecosystem combination if a threat did not occur in a grid cell (threat intensity = 0), or if the threat had an effect score of 0 for the ecosystem in question. The more high-intensity threats that occurred in a grid cell, and the more vulnerable the ecosystem in that cell was to those threats, the greater the cumulative impact score.

After calculating *CI*^*s*^ for all eight ecosystem types and for each of the three effect score scenarios (*s*), we merged the seven benthic ecosystem cumulative impact layers into a single raster for each scenario, with 250 × 250 m cell size (Fig. 1). The cumulative impact layers for the pelagic ecosystem were assessed separately. We summarised cumulative impact across the entire spatial range of each ecosystem type by generating density plots and calculating mean values for cumulative impact from all grid cells classified as each ecosystem type.

We also investigated the spatial consistency in cumulative impact results across all three scoring scenarios (best-case, most-likely and worst-case), highlighting spatially consistent results as being the most robust outcomes. Spatial consistency was assessed using percentiles, where grid cells that consistently scored low (≤20^th^ percentile) or high (≥80^th^ percentile) for cumulative impact under *all three scenarios* were classified into the ‘least-impacted’ and ‘most impacted’ zones respectively. This assessment was done separately on the benthic (all seven benthic ecosystem classes combined) and pelagic ecosystems. By virtue of the methodology (based on percentiles of the cumulative impact scores), a maximum of 20% of the area of each of the two broad scale ecosystem types (benthic and pelagic) could be classified into the ‘least’ and ‘most’ impacted zones; the higher the percentage value, the greater the spatial consistency across scoring scenarios. However, note that the when the ‘least’ and ‘most’ consistently impacted zones for the total benthic area of the Gulf were further split into the seven benthic ecosystem classes, the division of ‘most’ and ‘least’ impacted cells was not equal across the classes (i.e. some ecosystem classes were more frequently classified as ‘least’ or ‘most’ consistently impacted).

We calculated the total exposure (area) of each threat by summing the area it covered, at any intensity level >0, across the entire Gulf. We also ranked the threats using the sum of ‘most-likely’ cumulative impact scores from all grid cells that each threat occurred in. This ranking was done separately for the benthic (n = 7) and pelagic (n = 1) ecosystems, as these overlapped (Supplementary Figures S1 & S2). We ran sensitivity analyses to explore the influence of each threat and the effect scores on the most-likely scenario results; these are detailed in the Supplementary material (section S2.2).

### Measuring ‘knowledge-based uncertainty’

Our method for incorporating self-assessed ‘knowledge-based uncertainty’ into the results involved using the best-case and worst-case scenario effect scores to generate uncertainty bounds around the most-likely effect score. This was done by adding random errors to the average most-likely effect score for each ecosystem-threat pair, to simulate uncertainty. We compared three different methods for simulating uncertainty:

Method 1: Based on Stock and Micheli’s ‘assumed expert uncertainty method’^19^ given in Eq. 2, where *µ*_*i*,*j*_ is the average most-likely effect score for each ecosystem-threat pair, ε_*i*,*j*_ is a random number drawn from a uniform distribution bounded by 0.5 * the maximum effect score (in our study this was 8).

2 $${\hat{\mu }}_{i,j}={\mu }_{i,j}+{\varepsilon }_{i,j}$$

Method 2: An adapted version of Stock and Micheli’s method (see method 1 above), where errors were drawn from a uniform distribution with bounds defined by the most-likely score plus or minus up to half of the range between the best-case and worst-case scores (Eq. 3).

3 $${\hat{\mu }}_{i,j}={\mu }_{i,j}+{\varepsilon }_{k,l}$$

where *µ*_*i*, *j*_ is defined as for Eq. 1, ε_*k*, *l*_ is a random number drawn from a uniform distribution bounded by 0.5 * the difference between the best-case and worst-case scenario effect scores for ecosystem-threat pair (*i*, *j)*.

Method 3: Errors were drawn from a beta distribution, which is a distribution well-suited to representing uncertainty around expert elicited data^20^. The beta was parameterised by scaling the mean most-likely expert score for each habitat and threat combination using the mean best-case and worst-case scores, such that it fell within the range of 0 and 1: (most-likely − worst-case)/(best-case − most-likely). The alpha (α) and beta (β) parameters for the beta distribution were then calculated as described in Eqs 4 and 5 respectively:

4 $$\alpha =(((1-{\mu }_{i,j})\div{\sigma }^{2})-(1-{\mu }_{i,j}))\times {{\mu }_{i,j}}^{2}$$

5 $$\beta =\alpha \times ((1\div{\mu }_{i,j})-1)$$

where *µ*_*i*,*j*_ is the scaled average most-likely effect score for each ecosystem-threat pair and σ^2^ (variance) is fixed at 0.2. After back-transformation, this procedure produced unimodal sampling distributions bounded by the best- and worse-case effect score, and with mean equal to the most-likely effect score.

We used each of the three methods for uncertainty simulation to generate 1000 new effect scores for each ecosystem and threat pair. These were then used to parameterise 1000 iterations of the spatial cumulative impact assessment, thus simulating a plausible range of cumulative impact scores that attempted to account for knowledge-based uncertainty in three different ways. We summarised the results of the knowledge-based uncertainty simulations by calculating a mean and standard deviation from all 1000 iterations of cumulative impact for each ecosystem and for each of the three uncertainty methods.

### Data availability

The spatial datasets generated and analysed during this study are available from the Figshare repository. These data layers include the Spencer Gulf benthic and pelagic ecosystem layers (10.6084/m9.figshare.5047798.v1), all threat intensity layers (10.6084/m9.figshare.5047786.v1), and the cumulative impact score layers for the three expert scoring scenarios for both benthic and pelagic ecosystems (10.6084/m9.figshare.5047774.v1). The R scripts used for analyses during the current study are available from the corresponding author on reasonable request.

## Results

### Ecosystem and threat mapping

The eight dominant ecosystems of Spencer Gulf and their areal coverage are shown in Table 2 (for map see Supplementary Figure S1). The spatial exposure and spatial intensity (see glossary in Table 1) of each threat varied throughout Spencer Gulf, exposing some ecosystems to a greater number of threats than others (Table 3, Supplementary Figure S4). Mangroves had the highest average exposure to multiple threats throughout their spatial range, although seagrass and subtidal rocky ecosystems had the highest maximum threat exposure at any single point (Table 3). The pelagic ecosystem had the lowest threat exposure, both on average across its whole range and at any single location (Table 3).

**Table 2:** Area of the eight, mapped broad-scale ecosystems in Spencer Gulf.

Ecosystem class	Areal extent (km^2^)
Pelagic (water column in all sub-tidal areas)	29370
Subtidal soft sediment (includes invertebrate, rhodolith and sparse algal communities)	14552
Seagrass (intertidal and subtidal)	7423
Subtidal rocky (including algal forest and rocky reef communities)	1563
Saltmarsh	507
Intertidal soft (un-vegetated soft substrate)	382
Mangrove	87
Intertidal rocky (hard substrate)	16

**Table 3:** Summary statistics for threat exposure, based on spatial overlap between threats (N = 32) and ecosystems (N = 8). Values were calculated using all grid cells within each ecosystem class.

Ecosystem class	Summary statistics (per grid cell)		
	Min N threats	Maximum N threats	Mean N threats (std dev)
Intertidal rocky	6	16	8.06 (1.69)
Intertidal soft	6	19	8.14 (1.54)
Mangrove	6	16	9.99 (1.69)
Saltmarsh	6	15	8.49 (1.38)
Seagrass	5	20	7.59 (1.63)
Subtidal rocky	5	20	7.81 (1.63)
Subtidal soft	5	19	7.46 (1.11)
Pelagic	3	12	5.75 (1.26)

### Spatial cumulative impact assessment (standard approach, ‘most-likely’ scenario)

**Figure 2:**
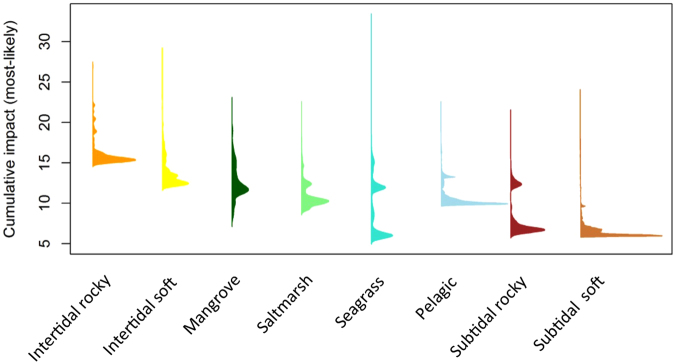
Density curves of the ‘most-likely’ (unscaled) cumulative impact score for each ecosystem in Spencer Gulf. The curves are generated using the cumulative impact scores from all grid cells classified as each ecosystem type.

**Figure 3:**
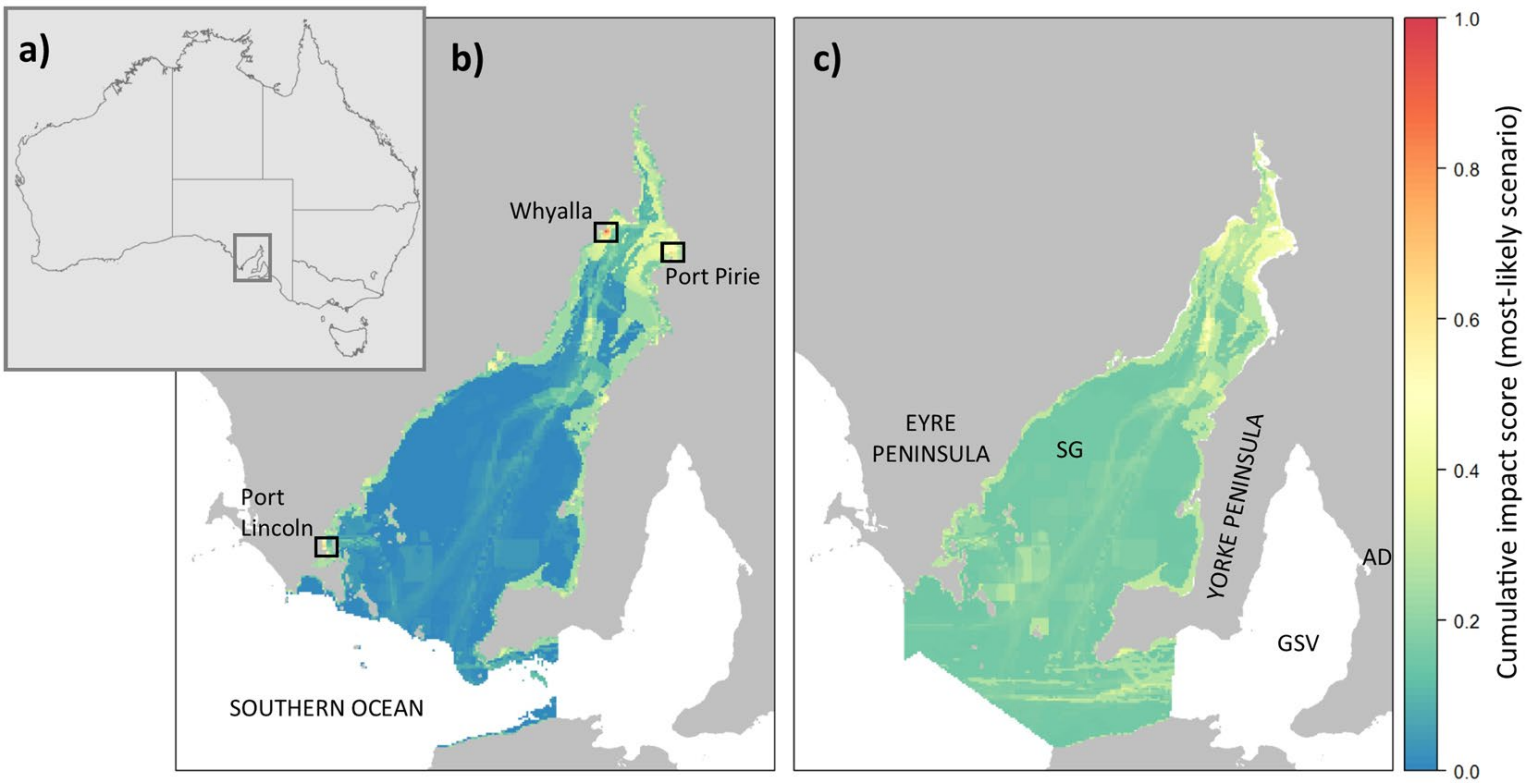
(**a**) Overview map of Australia with Spencer Gulf region shown by rectangle. Scaled cumulative impact maps for (**b**) benthic and (**c**) pelagic ecosystems in Spencer Gulf, based on the ‘most-likely’ scenario expert scores. Annotations: SG = Spencer Gulf, GSV = Gulf St Vincent, AD = Adelaide. Boxed areas in map (**b**) show location of population centres or industrial areas, finer-scale maps for these areas are supplied in the Supplementary material (Supplementary Figure S6). Maps were produced using R statistical software (version 3.3.1; https://www.r-project.org) and the packages raster^36^, rgdal^37^, sp^35^ and rasterVis^38^.

Seagrass ecosystems had the greatest cumulative impact score (>30) at a single location (Fig. 2), whereas the intertidal rocky ecosystem had the highest average cumulative impact score (most >15) across its entire spatial range (Fig. 2). Spencer Gulf’s intertidal and supratidal ecosystems were more likely to be exposed to higher levels of cumulative impact than subtidal and pelagic ecosystems (Figs 2, 3b,c). Cumulative impact score density curves for four ecosystems (saltmarsh, seagrass, pelagic and subtidal rocky) showed bimodality, indicating they had patches of both low cumulative impact and moderate to high cumulative impact (Fig. 2). Mangroves notably differed from all other ecosystems because the most common cumulative impact scores recorded for this ecosystem occurred in the middle of the score range, as opposed to occurring close to the minimum score (as was found for all the other ecosystems, Fig. 2).

For both pelagic and benthic ecosystems, the greatest cumulative impacts occurred in areas close to the coast, especially near major industrial developments and towns (e.g. Whyalla, Port Pirie and Port Lincoln, black outlines in Fig. 3b & Supplementary Figure S6). There were also concentrations of cumulative impacts for the pelagic ecosystem around the shipping channel in the middle of the northern Gulf, as well as in fishing and aquaculture areas in the south-central Gulf (Fig. 3c).

Our analysis based on the most-likely expert effect scores found that the top five threats for both benthic and pelagic ecosystems were pollution-related; including point source pollution, such as oil spills and nutrient inputs, and climate change threats resulting from global CO_2_ emissions (Table 4 & Supplementary Table S3).

**Table 4:** Summary statistics of impact scores for the top-five threats to benthic and pelagic ecosystems in Spencer Gulf, based on the experts’ most-likely effect scores. Summary values (min, max, median, mean and standard deviation) were calculated from all grid cells classified as each ecosystem type (all benthic ecosystem cells were grouped for this analysis). Sum = the total impact score attributable to each threat across all grid cells. Exposure = the total area of Spencer Gulf covered by the threat at any intensity level > 0. Images showing the exposure and intensity of each threat layer are provided in Supplementary Figure S3. R^2^ values were calculated using jack-knife sensitivity testing (see Supplementary material section S2.2.2) and indicate the influence of each threat layer on the final (most-likely scenario) cumulative impact scores. In this context, lower R^2^ values indicate a larger influence on the outcome (i.e. a greater difference after the threat was removed from the assessment). The full table of threat rankings is available in the online supplementary material (Supplementary Table S3).

	Exposure (km^2^)	Impact score summary:						R^2^
		Min	Max	Median	Mean	SD	Sum	
**Threat layer**		**Benthic ecosystems (combined) impact scores**						
Climate change: hot weather events	5190.3	<0.1	4.25	0.00	0.73	1.46	271264	0.552
Climate change: extreme rainfall events	30361.2	0.00	2.88	0.00	0.42	0.83	157525	0.743
Pollution: oil	3175.4	0.00	4.17	0.00	0.22	0.67	81049	0.893
Pollution: nutrients	3614.4	0.00	4.73	0.00	0.07	0.25	26843	0.957
Climate change: increased average temperature	30361.2	1.83	4.00	2.85	3.05	0.31	1133269	0.966
**Threat layer**		**Pelagic ecosystem impact scores**						
Climate change: ocean acidification	30361.2	3.75	3.75	3.75	3.75	0.00	1666710	0.669
Climate change: increased average temperature	30361.2	3.33	3.33	3.33	3.33	0.00	1481372	0.706
Climate change: extreme rainfall events	30361.2	2.75	2.75	2.75	2.75	0.00	1222254	0.757
Climate change: hot weather events	5190.3	0.00	3.42	0.00	0.41	1.11	180800	0.951
Pollution: oil	3175.4	0.00	3.84	0.00	0.24	0.73	107722	0.971

### Knowledge-based uncertainty – simulation of uncertainty bounds for the ‘most-likely’ scenario scores

**Figure 4:**
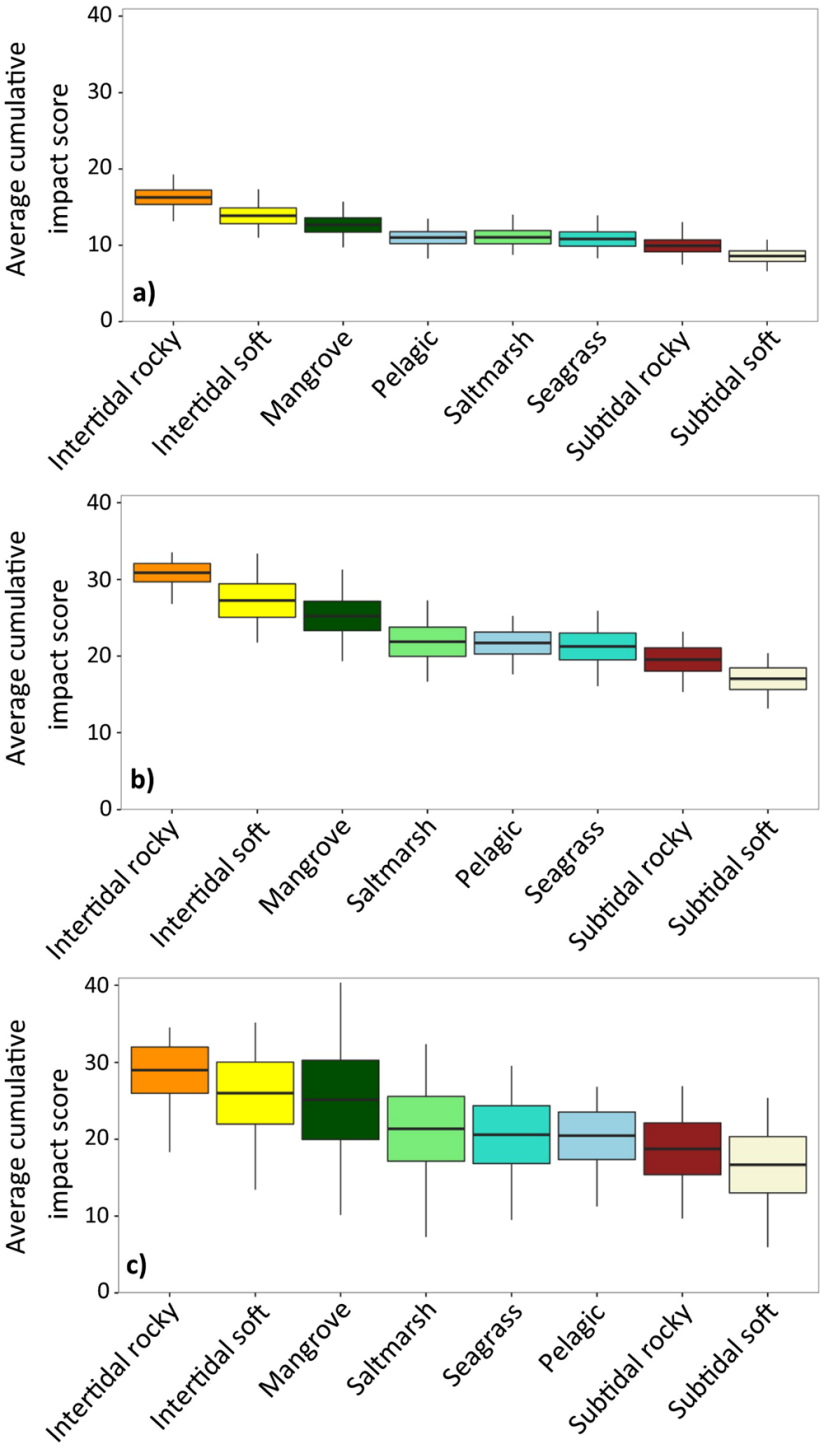
Summary plots of spatial cumulative impact scores from simulations that accounted for knowledge-based uncertainty. For each of 1000 simulations some random error was added to the expert effect scores for each ecosystem and threat combination and the adjusted scores were used to calculate cumulative impact. (**a**) Using the expert’s self-assessed uncertainty data, simulated values were drawn from a beta distribution centred on the most-likely score and bounded by the best-case and worst-case scores (Eqs 4 and 5). For (**b**) and (**c**), simulated random errors were drawn from a uniform distribution based on (**b**) our expert self-assessed uncertainty method (Eq. 3) and (**c**) assumed uncertainty method proposed by Stock and Micheli^19^ (Eq. 4). In all panels the bold horizontal line shows the average cumulative impact score for each ecosystem class from 1000 simulations, box extents show the standard deviation of this average and vertical lines extend to the minimum and maximum average cumulative impact per ecosystem recorded across all 1000 simulations.

The cumulative impact rankings of the eight Gulf ecosystems remained broadly similar across the three approaches to incorporating expert uncertainty; although note the switch in positions of pelagic, saltmarsh and seagrass ecosystems in Fig. 4a–c. Both of the self-assessment uncertainty approaches (Fig. 4a,b) resulted in smaller standard deviations compared to the assumed uncertainty approach (Fig. 4c). Assuming our survey method appropriately captured the experts’ uncertainty about the effect of threats on ecosystems, this outcome suggests that results were better constrained when we used the data collected from the experts to quantify knowledge-based uncertainty. However, there is a risk that linguistic uncertainty introduced during the expert survey may have caused the elicited bounds to be unrealistically narrow. Future surveys should follow the advice in Regan *et al*.^40^ and Carey *et al*.^41^ to reduce the risk of linguistic uncertainty in expert elicitation.

Using a uniform distribution to simulate uncertainty resulted in higher cumulative impact scores (Fig. 4b,c) and often did not adequately capture the experts’ most-likely scores. This can be seen when comparing the values from the boxplots (Fig. 4b,c) with the density plots of most-likely scores from the entire spatial range of each ecosystem (Fig. 2). This results from the uniform distribution assumption of symmetrical uncertainty around the most-likely score, which we found not to be the case in most instances (See Supplementary material S2.4 and Supplementary Figure S7). The beta distribution method for modelling uncertainty allowed asymmetrical bounds around the most-likely score (see Supplementary material S2.5, Supplementary Figure S8) and thus resulted in a better representation of both the most-likely expert effect scores and the self-assessed uncertainty ranges.

### Self-assessed knowledge-based uncertainty – spatial consistency

**Figure 5:**
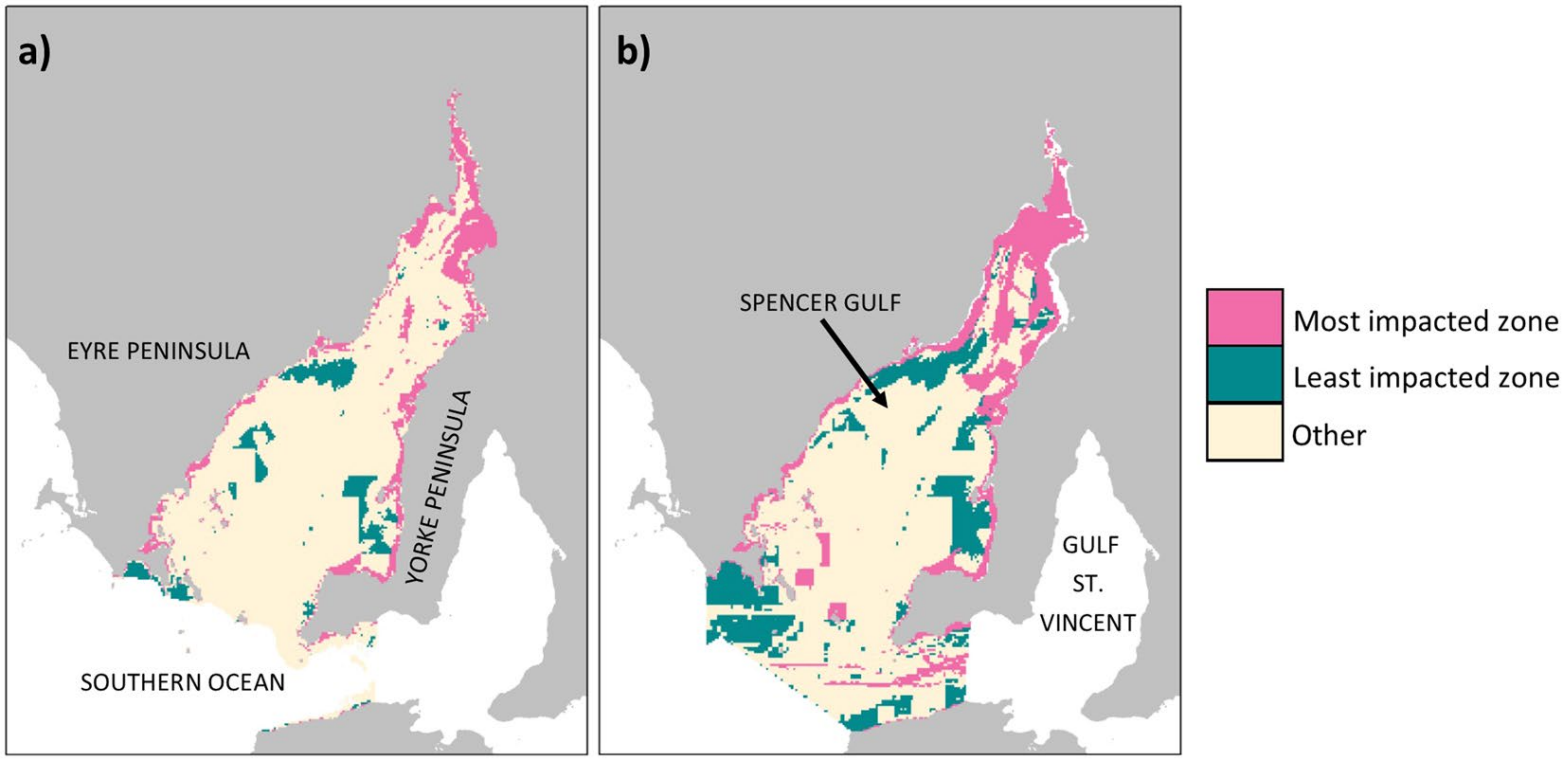
Maps showing the grid cells that were consistently the ‘most impacted’ (≥80^th^ percentile, pink) or ‘least impacted’ (≤20^th^ percentile, green) across all three effect score scenarios (best-case, most-likely and worst-case) for (**a**) benthic and (**b**) pelagic ecosystems. Tan coloured areas show pixels that were not consistently in either the upper or the lower percentile groups (i.e. had scores between the 20^th^ and 80^th^ percentile in at least one scoring scenario). Maps were produced using R statistical software (version 3.3.1; https://www.r-project.org) and the packages raster^36^, rgdal^37^, sp^35^ and rasterVis^38^.

Using the best-case, most-likely and worst-case scenario effect scores from the expert survey^22^, we undertook three versions of the spatial cumulative impact assessment and explored spatial consistency across all three results; identifying areas consistently classified as ‘most’ or ‘least’ impacted, based on a percentile method. The ‘most impacted’ zones for both the benthic and pelagic broad scale ecosystems tended to be in the northern Spencer Gulf and coastal areas (Fig. 5). The ‘least-impacted’ zones (containing cells with consistently low cumulative impact scores) were generally found in southerly parts of the Gulf and areas with moderate depth (Fig. 5).

Values close to 20% for the total coverage of the benthic or the pelagic ecosystems within each impact zone indicate high consistency in the cumulative impact scores across all three scoring scenarios (Table 5). Whereas low percentage values indicated that there was little overlap between the areas classified as ‘least’ or ‘most’ impacted across all three scoring scenarios. There was greater consensus (indicative of greater confidence) for the pelagic ‘most’ (19.3%) and ‘least’ (16.9%) impacted zones under the three scoring scenarios, compared to the *total* benthic ecosystem impact zones (calculated based on the total area of all seven classes of benthic ecosystem), which were 13.3% and 6.8% respectively.

**Table 5:** Percentage and area (km^2^) of each broad scale ecosystem type (benthic and pelagic), and each benthic ecosystem class, which scored ≤20^th^ percentile (‘least impacted’) and ≥80^th^ percentile (‘most impacted’) for cumulative impact under *all three* effect score scenarios (best-case, most-likely and worst-case). The *benthic ecosystem total* shows the area of the ‘most’ and ‘least’ impacted zones as a percentage of the entire area of all benthic ecosystems in the Gulf

**Ecosystem**	***Consistently most-impacted***		***Consistently least-impacted***	
	**Percentage**	**Area (km** ^**2**^ **)**	**Percentage**	**Area (km** ^**2**^ **)**
Pelagic ecosystem	19.3	5679.8	16.9	4959.0
Benthic ecosystems (total)	13.3	3269.0	6.8	1677.7
Benthic - Intertidal Soft	100.0	382.4	0.0	0.0
Benthic - Mangrove	87.6	76.1	0.0	0.0
Benthic - Saltmarsh	41.8	211.7	0.0	0.0
Benthic - Seagrass	25.6	1901.7	0.0	0.0
Benthic - Subtidal Rocky	26.4	412.3	0.0	0.0
Benthic - Subtidal Soft	2.0	284.8	11.5	1677.7

.

We further investigated the impact zones as a function of which specific ecosystem class they covered. The full spatial extent of the intertidal rocky and intertidal soft areas of the Gulf were encompassed in the consistently ‘most-impacted’ zone for benthic ecosystems. The two ecosystem classes with the largest areas classified as ‘most-impacted’ were seagrass (1901.7 km^2^) and pelagic (5679.8 km^2^) (Table 5). The ecosystem with the lowest percentage of its area classified as ‘most impacted’ was subtidal soft (2%), although this ‘small’ percentage equates to 284.8 km^2^, which is more than 18 times the total size of the intertidal rocky ecosystem (Table 5). There were only two ecosystem classes with any part of their area consistently classified as ‘least-impacted’; these were subtidal soft (1677.7 km^2^) and pelagic (4959 km^2^), which was also the ecosystem class with the greatest percentage of its area (16.9%) within the consistently ‘least-impacted’ zone (Table 5). Six of the seven benthic ecosystem classes (intertidal rocky, intertidal soft, mangrove, saltmarsh, seagrass and subtidal rocky) had none of their extent in the benthic consistently ‘least impacted’ zone (Table 5).

## Discussion

Lack of data drives the use of expert knowledge in spatial cumulative impact assessments for all environments^6,42–45^. However, there are drawbacks to using expert knowledge as a surrogate for quantitative empirical data, a key one being knowledge-based uncertainty^16^ and the difficulty in accounting for it^21^. We demonstrate that this source of uncertainty can affect spatial cumulative impact assessments, and their interpretation, particularly regarding the level of confidence to ascribe to mapped outputs. Exploration of the impact of knowledge-based uncertainty on assessment results is important for testing the reliability of cumulative impact assessment methods and outputs^19,46,47^. It also improves the transparency of the assessment process and highlights the most robust results, which is critical if the outputs are to be used for identifying knowledge gaps, directing funding, monitoring, or informing management actions^6,8,18^.

We requested that experts provided an effect score range using scoring scenarios^22^, as is commonly recommended in the literature on expert elicitation^6,15,16,19^. This led to upper and lower bounds (best-case & worst-case scenarios respectively) around the most-likely effect scores. There is a risk that linguistic uncertainty (uncertainty in language) related to each expert’s own interpretation of the terms ‘best-case’, ‘worst-case’ and ‘most-likely’, may have introduced uncertainty into our survey results^40,41^. We attempted to avoid this issue by providing a definition of these terms in the survey reference sheet (a copy of which can be found in the Supplementary material of Doubleday *et al*.^22^).

In addition, experts may be over-confident when assessing their own uncertainty^15^, but we cannot test this without knowing the ‘correct’ effect score for each ecosystem and threat pair, which would require validation with empirical data that are not currently available. Our approach required additional time and effort from both the experts and the researchers, because three effect scores were needed for each ecosystem and threat combination. However, we propose that through thoughtful survey design and expert selection^22^, these time costs can be reduced. We also demonstrate that these additional time costs are balanced by the benefits of gathering these valuable data on uncertainty, which increase the confidence in, and utility of, the assessment outputs.

Our spatial cumulative impact assessment for Spencer Gulf found that intertidal and near-shore pelagic/subtidal benthic ecosystems, especially those around heavy industry and population centres, consistently scored above the 80^th^ percentile for cumulative impact across all three scoring scenarios. The intertidal rocky and subtidal soft ecosystems respectively had the highest and lowest average cumulative impact across their entire spatial ranges. The ecosystem classes all ranked similarly for cumulative impact under the different methods for simulating knowledge-based uncertainty; however, the absolute values of average cumulative impact for each ecosystem changed substantially depending on the assumptions of the distribution used to simulate uncertainty. Using the beta distribution to simulate uncertainty around the most-likely expert scores resulted in the lowest average cumulative impact scores and the smallest amount of variation. The beta distribution was centred on the most-likely score and bounded by the best-case and worst-case scores; meaning it accurately reflected these three information points provided by the experts, which represented plausible ranges for the impact of threats on ecosystems in the Gulf. Conversely, the method we used to parameterise the uniform distribution (based on that of Stock and Micheli^19^) used only the best-case and worst-case scores to generate error values and assumed that uncertainty was symmetrical around the most-likely score. This assumption resulted in simulated ranges of effect scores that could exclude the experts most-likely effect score, in cases when the best-case and worst-case scores were not equidistant from the most-likely score. The plausible score ranges that we collected from the experts were frequently not centred on the most-likely score; meaning they could not be appropriately represented by a uniform distribution.

If self-assessed knowledge-based uncertainty data are not available (as is the case in many previous studies), the Stock and Micheli method^19^ may be a conservative alternative for retrospectively accounting for knowledge-based uncertainty in cumulative impact assessment results (being based on *a priori* assumptions)^19^. However, it must be acknowledged that the bounds set on the distribution of uncertainty are arbitrary and the method can lead to over-inflation of both the average cumulative impact scores and the amount of uncertainty around them, if expert uncertainty is not symmetrical around the given score.

In addition to our primary focus on knowledge-based uncertainty, we also aimed to deliver useful outputs for the sustainable management of Spencer Gulf. We found that high cumulative impacts in coastal areas were driven both by the greater effect scores for threats to shallow and intertidal ecosystems^22^ and the greater spatial exposure and intensity of sea- and land-based threats in coastal areas. Similar patterns of high cumulative impact in shallow coastal areas have also been shown in other studies at both global-^1,2^ and regional-scales^12,17,44,48,49^.

We expected that most of each ecosystem would experience relatively low-level cumulative impact, with a small proportion having moderate to high cumulative impact scores. This pattern was borne out in the cumulative impact density curves for the intertidal rocky, intertidal soft and subtidal soft ecosystems. However, we found that the density curves for saltmarsh, seagrass, pelagic and subtidal rocky ecosystems were bimodal, indicating that considerable patches of these ecosystem classes experienced moderate to high cumulative impact. The patches experiencing higher cumulative impact were predominantly in northern Spencer Gulf and coastal areas, where cumulative impact scores were consistently higher than in the southern or deeper, central areas of the Gulf. Mangroves differed from other ecosystems because the peak in cumulative impact occurred in the middle of the score range, rather than being skewed to the lower end of the scale. This suggests that most mangroves throughout Spencer Gulf are at least moderately impacted by cumulative threats.

High cumulative impacts on Spencer Gulf’s coastal ecosystems are likely to affect the delivery of key ecosystem services from these areas^50^, including recreation, provision of fish habitat and nursery areas, water purification, coastal protection and carbon sequestration^51–53^. A reasonably large proportion (19.3%) of the Gulf’s pelagic ecosystem fell within the consistently ‘most-impacted’ zone: 5680 km^2^, with a smaller proportion (3269 km^2^ and 13.3%) of the total area of Spencer Gulf’s benthic ecosystems found to be consistently at risk of high cumulative impact (i.e. in the ‘most-impacted’ zone). This suggests that there is greater certainty about the risk of cumulative impacts to the pelagic ecosystem than the benthic ecosystem; shown by more consistent results across the three certainty scenarios for the pelagic ecosystem.

Our results indicate that Spencer Gulf’s benthic ecosystems are relatively unaffected by human activities compared to other multi-use aquatic systems that support industrial and recreational activities, such as the Mediterranean and Black Sea^48^, the Baltic Sea^17^ and the Great Lakes in North America^44^. This assertion is based on qualitative visual assessment of the figures in these papers, which indicate greater cumulative impact scores over larger areas compared to our results for Spencer Gulf (it is difficult to make this comparison quantitatively because the cumulative impact scores in these papers are not described using directly comparable percentile metrics). This apparent difference in the extent and severity of cumulative impacts is likely to be driven by greater population densities (with associated development and industrialisation) in these regions, compared to the Spencer Gulf region. The areas surrounding the Mediterranean Sea and the Great Lakes region of the USA have respective averages of 20–1000 ^54^ and 91.5 ^55^ people per km^2^, compared to just 1.62 people per km^2^ in South Australia (with a range of 0.1–10 people per km^2^ in areas immediately surrounding Spencer Gulf)^56^. This current, comparatively low impact of human activity in Spencer Gulf provides an important opportunity for early intervention through the development of an integrated management approach for the region. Adopting such a framework now, before broad-scale environmental degradation becomes a widespread issue, may avoid the need for ecosystem restoration later.

According to our semi-quantitative assessment for the eight assessed marine ecosystems in Spencer Gulf, the top five threats were related to either point source pollution from oil and nutrients, or climate change. In a recent update^2^ to their earlier global analysis^1^, Halpern *et al*. found that the impacts of climate change have increased over the last 5 years in 66% of the global ocean. These findings are particularly concerning as impacts from global climate change are difficult to manage at a local, or even regional level^57^. This makes it even more important to reduce other high-impact threats that can be managed locally, e.g. point source pollution in Spencer Gulf. This will contribute to reducing overall pressures on marine ecosystems and potentially increase their resilience to climate change^58–60^.

All spatial cumulative impact assessments make a number of assumptions^6^, each of which may influence the results^19^. Our study highlights the effect that knowledge-based uncertainty can have on assessment results; the ease with which self-assessed data on uncertainty bounds can be collected; and how confidence in results can then be tested and reported by simulating bounds around average impact scores and focusing on the most consistent results from across all effect score scenarios. However, further uncertainty in our results may stem from assumptions we have made during the assessment process, which are common to other cumulative impact assessments^6^. We assumed that an ecosystem exposed to single, or multiple threats *would* be impacted; that the impact was linear; and that the impact of multiple threats was additive (rather than interactive, synergistic or antagonistic^61^). Other caveats include that we could not include pelagic/midwater line and net fishing in our assessment, because of the poor spatial resolution of these data for our study region. However, the impact of these types of fishing gear on ecosystems (as opposed to specific species^62^) is expected to be minimal, especially when compared to other fishing gears with greater impact on the seascape, such as trawling^22,63^, which was included. In addition, the available climate change data had poor spatial resolution, meaning that these threats were often treated as ubiquitous (Supplementary Figure S3e). We were not able to include three particularly vulnerable ecosystems (rhodolith beds^31,32^, sponge gardens^30,64^ and native shellfish beds^33^) because of a lack of spatial data leading to uncertainty around their location and spatial extent throughout the Gulf. The unavailability of these data identify clear knowledge gaps based around a lack of well-resolved spatial information on ecosystems, human activities and impacts of climate change, as well as a need for more empirical studies on the impact of various threats, including the likely complex interactions between multiple threats^65^. This lack of data also highlights that knowledge-poor areas or ecosystems are often those that are most vulnerable and difficult to evaluate.

Our method enables new or updated spatial data layers to be included as they become available; particularly if they relate to the distribution of ecosystems or threats that were included in the original expert elicitation survey^22^. Future work should involve validation of our predictions of cumulative impact using *in-situ* data on ecosystem condition and the inclusion of potentially positive human activities, such as marine parks and no-take zones.

## Conclusion

Our study of Spencer Gulf, based in part on expert-elicited data, showed that the greatest risk of cumulative impacts to the eight assessed coastal and marine environments occurred in intertidal and shallow subtidal ecosystems, particularly in the northern Gulf. This was consistent across all expert score uncertainty scenarios and was due to: 1) greater concentrations of high-intensity threats in coastal areas, especially close to heavy industry and population centres; and 2) higher effect scores for these ecosystems (indicative of their greater vulnerability to threats), particularly climate change and localised pollution. Overall, however, only a relatively small proportion of Spencer Gulf’s marine ecosystems appear to be exposed to high risks from cumulative impact when qualitatively compared to published studies for other locations. This is a positive outcome, and means our results can be used to support conservation and proactive, protective management, rather than exclusively highlighting priority areas for restoration.

We show that uncertainty associated with expert knowledge can lead to uncertainty in the outputs of spatial cumulative impact assessments. However, we recognise the value of expert knowledge for assessments of data-poor regions and environments (commonly the case for marine systems). Therefore, we demonstrate a straight-forward method for capturing uncertainty in expert knowledge, which is simply to ask the experts for effect score *ranges* indicative of their uncertainty, when assessing the effect of threats to ecosystems. This approach enables knowledge-based uncertainty to be accounted for and the most certain results to be identified.

## Electronic supplementary material

Supplementary information
