# Supplementary material for: Capturing expert uncertainty in spatial cumulative impact assessments
Source: Sci Rep. 2018 Jan 23;8:1469. doi: 10.1038/s41598-018-19354-6 (PMC5780512; doi:10.1038/s41598-018-19354-6)
Supplement: Supplementary file 1 — Supplementary information [file 41598_2018_19354_MOESM1_ESM.pdf]

Electronic Supplementary Material for Jones *et al.*:

'Capturing expert uncertainty in spatial cumulative impact assessments'

Alice R. Jones, Zoe A. Doubleday, Thomas A. A. Prowse, Kathryn H. Wiltshire, Marty R. Deveney, Tim Ward, Sally L. Scrivens, Phillip Cassey, Laura O'Connell, Bronwyn M. Gillanders.

S1: Methods

S1.1. Ecosystem mapping

We combined all available data on the locations of benthic ecosystems in Spencer Gulf. These layers included spatial polygons derived from aerial imagery, modelled predictions for seagrass cover through the Gulf[3] and sediment sample data[5] (Supplementary Table S1). The different data layers had varying native resolutions and confidence levels. This was dealt with by using a data hierarchy system where the best available data were given preference over other layers in areas with coverage from two (or more) layers. The hierarchy is shown in Supplementary Table S1 and was determined with advice from the creators and distributors of the data layers.

Supplementary Table S1: Data layer sources for marine benthic ecosystem mapping and their rank in the data hierarchy (based on resolution, reliability and date of data collection). DEWNR = South Australian Department for Environment, Water and Natural Resources.

Data layer (and link to metadata where available)	Source	Data collection year	Resolution	Rank
Saltmarsh and Mangrove habitats	DEWNR	2005	± 10 to 20 m	1
State marine benthic habitats	DEWNR	2004	± 10 to 20 m	2
National marine benthic habitats	DEWNR	1998	± 100 to 1000 m	3
Estuary habitats	DEWNR	2009	± 15 m	4
Modelled seagrass	[3]	NA	250 m	5
Seafloor surface sediment grab samples	[5]	Various, from 1990 onwards	NA	6

The data layers were merged according to the data hierarchy in Supplementary Table S1 and were then rasterised to a grid with ca. 250 m cell size (Supplementary Figure S1a). By combining multiple sources of data, we were able to generate a map with full coverage of the study area. However, combining data from different sources, with different resolutions and collected in different ways, can introduce uncertainty into the resulting ecosystem map. Uncertainty was assessed by looking for discrepancies between the ecosystem class attributed at the same location by different data layers. Uncertainty in ecosystem classification arose for three main reasons:

1. Discrepancies between the ecosystem classes attributed by different DEWNR data layers in areas where they overlapped. See dark grey areas in Supplementary Figure S1b. In these

areas, ecosystem type was designated using the highest ranked DEWNR ecosystem layer (see Supplementary Table S1 for hierarchy)

2. Discrepancies between a DEWNR ecosystem layer and the modelled seagrass layer. See light grey areas in Supplementary Figure S1b. In these areas, preference was given to the DEWNR data layers as per the hierarchy above (Supplementary Table S1).
3. No data available on the specific benthic ecosystem in an area of the Gulf (mostly in deeper, central and southern areas). These are the sections where we have inferred benthic ecosystem type based on biological communities and sediment type from benthic grab samples[5]. This area is shown by the light grey hashed zones in Supplementary Figure S1b. The locations of grab samples, along with their ecosystem class attribution are shown by the filled circles. Note that the ecosystem classes identified by O'Connell *et al.*[5] (based on biological attributes of the sediment samples) were translated to one of our pre-defined Spencer Gulf ecosystem classes as per the list below:
    - Dense seagrass meadows -> Seagrass
    - Rhodolith pavements -> Subtidal soft bottom
    - Patchy seagrass and sand flats -> Subtidal soft bottom
    - Muddy seafloors -> Subtidal soft bottom
    - Open gravel/sand plains -> Subtidal soft bottom

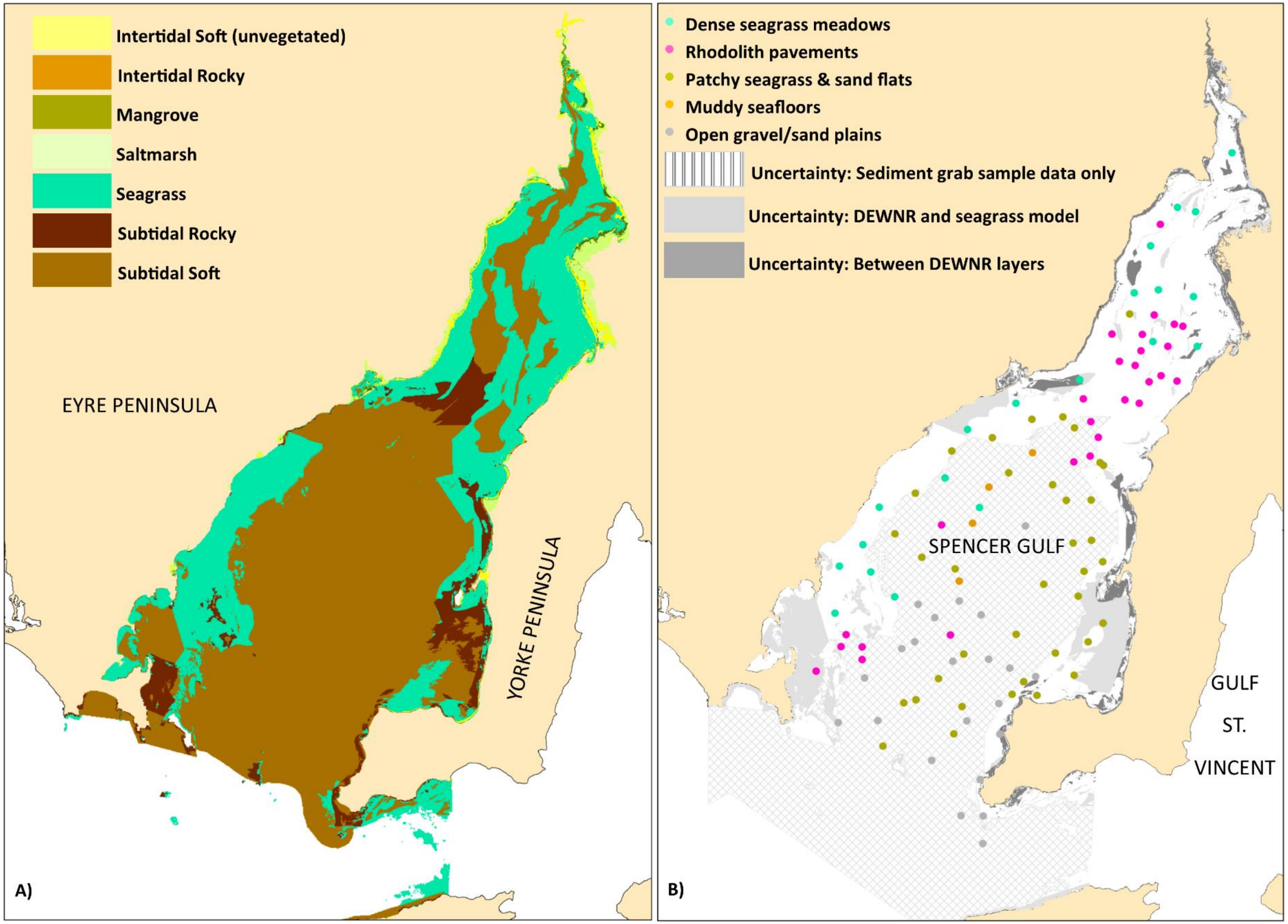

Supplementary Figure S1: A) Map of broad-scale marine benthic ecosystems in Spencer Gulf (coverage of the ecosystem map was determined by the combined extent of available ecosystem data layers). **B)** Areas of, and reasons for, uncertainty in benthic ecosystem class attribution and position of sediment grab samples with biofacies class attribution[5]. Maps were produced using R statistical software (version 3.3.1; https://www.r-project.org) and the packages raster[1], rgdal[2], sp[4] and rasterVis[6].

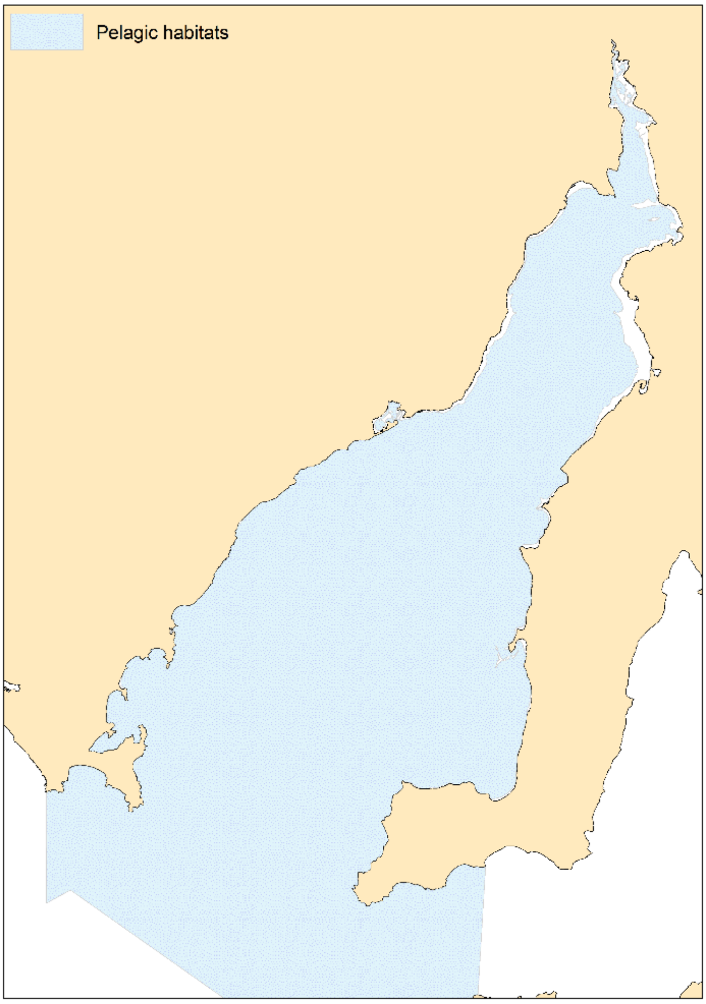

Supplementary Figure S2: Map of area defined as containing pelagic ecosystems (overlaying the benthic ecosystems shown in Supplementary Figure S1a). Pelagic ecosystems were defined as water column ecosystems, overlaying the seafloor in subtidal areas. Map was produced using R statistical software (version 3.3.1; https://www.r-project.org) and the packages raster[1], rgdal[2], sp[4] and rasterVis[6].

The Spencer Gulf benthic and pelagic ecosystem layers are downloadable from the Figshare data repository using the following link: https://figshare.com/s/adb72dfb903dfda58ede.

S1.2. Threat mapping:

32 threats were mapped throughout the Spencer Gulf study area using the best available data (see Supplementary Table S2 for details on the data used and Supplementary Figures S3 a-e for spatial intensity/exposure maps). The majority (n = 26) of the threats could be mapped by *spatial intensity*: A continuous score, between 0 and 1, which represents the relative intensity of a threat at any grid cell within the study area. The cell with the highest spatial intensity score (1) for each threat is the location where this threat occurs at the greatest intensity. For example, for lobster pot fishing, this would be the location with the highest average number of pots dropped. Six of the threats were mapped using a simpler, binary (0 or 1) measure of spatial exposure, which only represents the absence

(0) or presence (1) of a threat within a given grid cell and does not provide information on the relative intensity of the threat at the locations where it does occur.

All threats were mapped on a common raster grid with 250 x 250 m cell size, which involved either up or downscaling from the original resolution of the spatial data layers (see details in Supplementary Table S2) and assumes that a threat was evenly distributed within each of the native resolution grid cells or spatial polygons.

All threat data layers are available to download from the Figshare repository using the following link: https://figshare.com/s/8953f66d9a91f069ba01

Supplementary Table S2: List and brief description of the threat layers used in the cumulative impact assessment for Spencer Gulf. Threats marked with an * were mapped as a binary score (0 or 1) indicating presence or absence, because relative intensity values were not available. DEWNR = Department of Environment; Water and Natural Resources, PIRSA = Department of Primary Industry and Regions South Australia; DPTI = Department of Planning, Transport and Infrastructure South Australia; SARDI = South Australian Research and Development Institute. Maps of threat layers are shown in Supplementary Figures S3a – e.

Threat layer	Details
Acid sulphate soil	Spatial data provided by DEWNR. Intensity based on DEWNR risk classification.
Subtidal introduced marine species - encrusting and fouling	Data from PIRSA[7]: location of observations of introduced marine species from surveys and opportunistic sampling. Introduced species were classed into functional groups. Intensity for each functional group was based on the number of different species from that group recorded within a 5 x 5 km grid cell and interpolated to 250 x 250 m cell size. Note that only macroscopic pests and parasites (e.g. shell boring worms) were included – not microscopic pests, parasites or pathogens.
Subtidal introduced marine species - predatory and parasitic	
Subtidal introduced marine species - benthic filter feeding	
Intertidal introduced marine species - encrusting and fouling	
Intertidal introduced marine species - predatory and parasitic	
Intertidal introduced marine species - benthic filter feeding	
Coastal activities (e.g. walking, sunbathing, off-road vehicles, grazing).	Spatial land use data from DPTI and population data from Halpern *et al.*[8]. Intensity was based on a combination of the size of local populations and the distance from the nearest grazing land to each grid cell.
Coastal habitat modification (e.g. land reclamation, native vegetation removal, shoreline hardening)	Vegetation-type data from Department for the Environment. Intensity was based on the distance of each grid cell to the nearest modified habitat.

Threat layer	Details
Commercial harbours and ports (N = 5)	Spatial data provided by DEWNR. Intensity was based on the distance from each grid cell to each feature and weighted by the relative size of the features (or the annual average number of ship visits, in the case of the ports).
Jetties, wharves, breakwaters, slipways and moorings (N = 116)	
Marinas (N = 6), boat ramps (N = 54) and ferry terminals (N = 1)	
Commercial fishing: Southern rock lobster (pots)	Spatial data provided by PIRSA. Intensity was based on the annual average number of pot lifts per fishing area.
Commercial fishing: Prawn trawling	Spatial data provided by PIRSA. Intensity was based on the annual average trawl distance per fishing area.
Commercial fishing: Sardine fishery (seine net)	Spatial data provided by PIRSA. Intensity was based on the annual average number of nets deployed per fishing area.
Commercial fishing: Abalone (hand collection)	Spatial data provided by PIRSA. Intensity was based on the annual average hours dived in each fishing area.
Heavy metal contamination	Data from Gaylard[9]. Intensity was based on the volume of input and weighed by the distance from each grid cell to each input source.
Point source thermal pollution (N = 2)	
Boating (includes recreational activities, aquaculture and ecotourism).	Spatial data from PIRSA (aquaculture), DEWNR (recreational fishing), SARDI (ecotourism) and local dive clubs (locations of popular dive destinations). Intensity was based on total boating activity in each grid cell.
Point source inorganic nutrient pollution (nitrogen and phosphorus)	Data from Gaylard [9] and Halpern *et al.*[8]. Intensity was based on the volume of input and weighted by the distance from each grid cell to the input source.
* Aquaculture: oysters	Spatial data provided by PIRSA. Intensity was based on presence (1) or absence (0) of an active aquaculture operation in a grid cell.
* Aquaculture: mussels	
Aquaculture: finfish (including nutrient inputs)	Spatial data on the locations of sea cages was provided by PIRSA, scored as presence (1) or absence (0) of an active aquaculture operation in a grid cell. Modelled spatial data on ammonium levels was provided by from SARDI [10] and the area exposed to elevated levels was defined by cells where ammonium levels increased >15 % from baseline levels (and scaled according to the percentage increase). Intensity was based on a combined sea cage presence/absence and relative nutrient increase score.
Oil spill risk	Spatial data was sourced from the Halpern *et al.*[8] ocean pollution index spatial layer.

Threat layer	Details
Current shipping: low intensity (< 100 voyages p.a.)	Intensity was based on the number of shipping voyages (vessels > 300 gross tonnes) passing through each grid cell (using Automatic Identification System tracking data from 1st Aug 2013 – 31st July 2014).
Current shipping: moderate intensity (100 - 200 voyages p.a.)	
Current shipping: high intensity (> 200 voyages p.a.)	
* Ocean acidification	We did not find any fine-scale spatial data on how the impacts of these climate-change related threats vary throughout the Gulf, so they were mapped as ubiquitous (all grid cells were given a value of 1).
* Decreased annual rainfall	
* Extreme rainfall events	
* Global warming	
Hot weather events	Short-lived heat waves will impact shallower areas disproportionately, therefore this threat was limited to affecting only areas on land or < 10 m subtidal depth. All cells within this area were given a value of 1, and all other cells were given a value of 0.

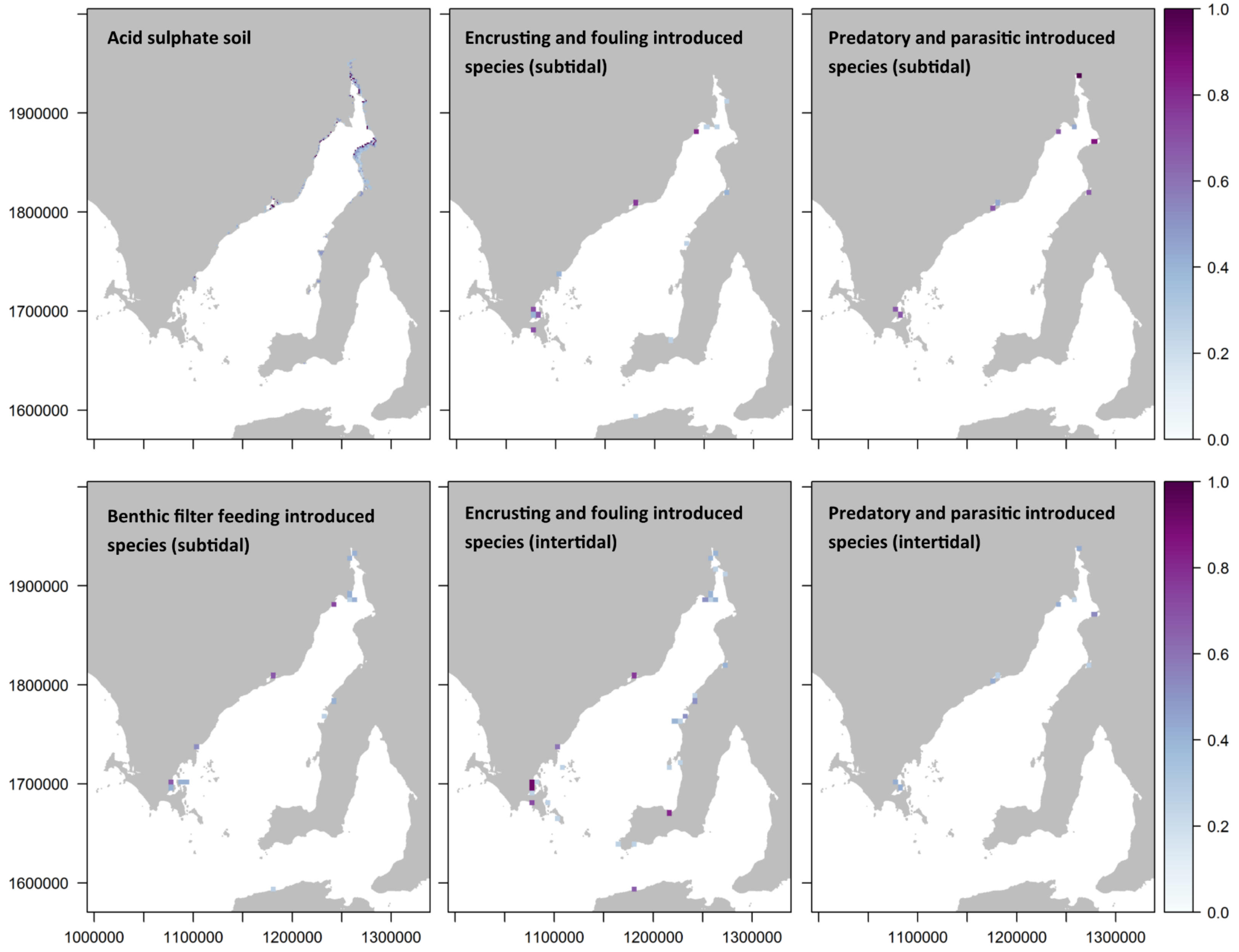

Supplementary Figure S3a: Threat spatial intensity maps. Values were scaled to between 0 and 1 and represent the relative intensity of a threat at any grid cell within the Spencer Gulf study area. For details on scaling method see glossary in Table 1 (main text). Maps were produced using R statistical software (version 3.3.1; https://www.r-project.org) and the packages raster[1], rgdal[2], sp[4] and rasterVis[6].

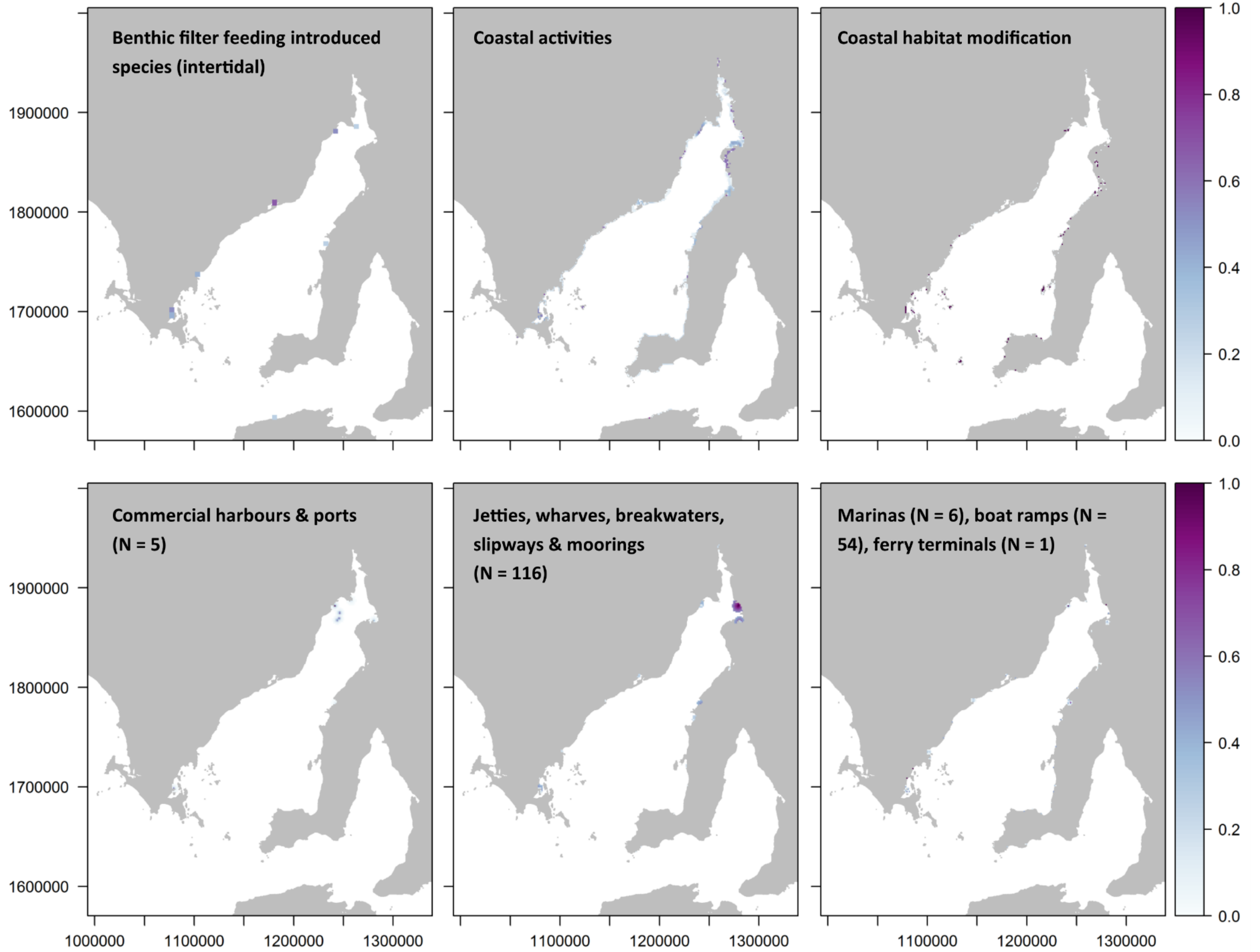

Supplementary Figure S3b: Threat spatial intensity maps continued. Values were scaled to between 0 and 1 and represent the relative intensity of a threat at any grid cell within the Spencer Gulf study area. For details on scaling method see glossary in Table 1 (main text). Maps were produced using R statistical software (version 3.3.1; https://www.r-project.org) and the packages raster[1], rgdal[2], sp[4] and rasterVis[6].

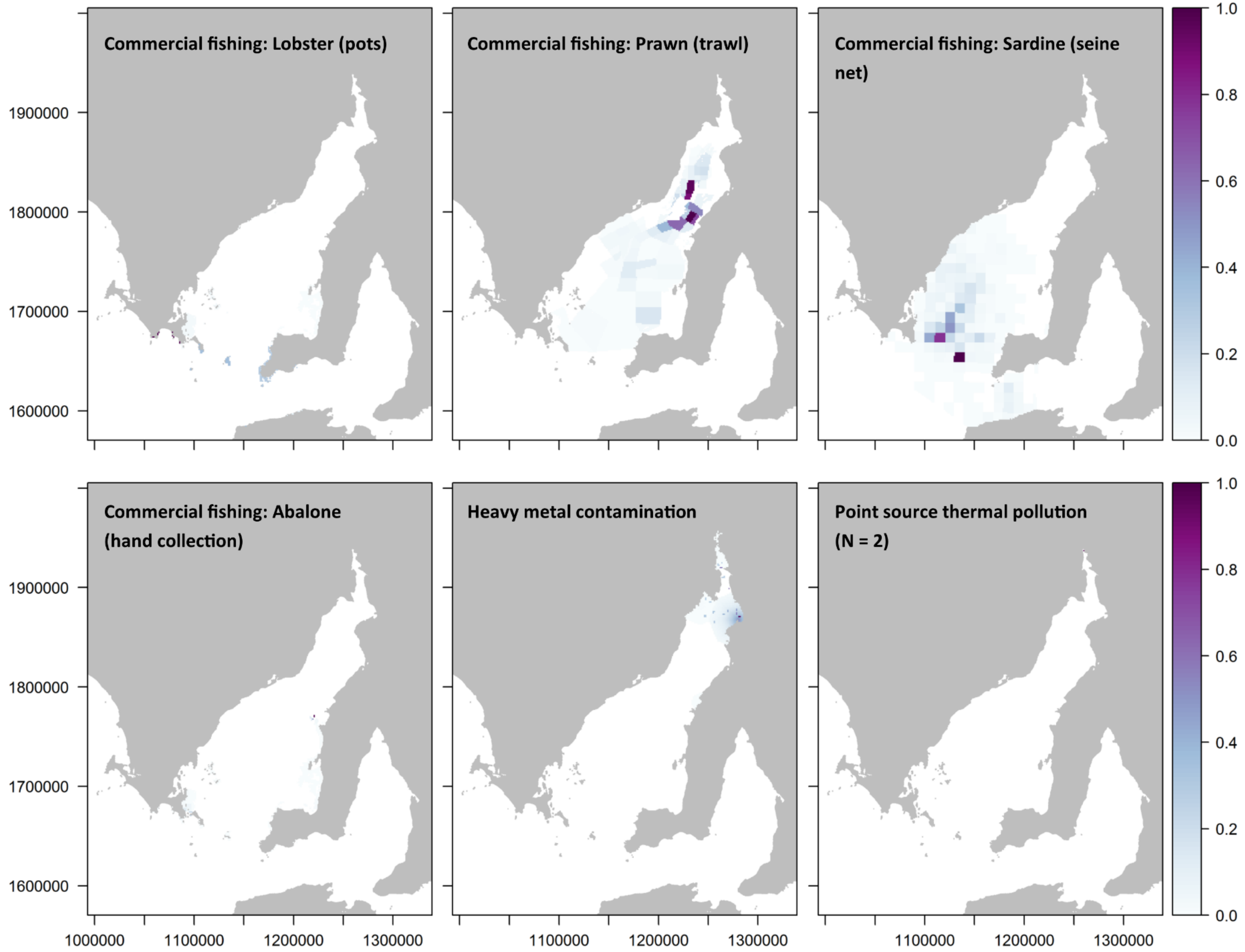

Supplementary Figure S3c: Threat spatial intensity maps continued. Values were scaled to between 0 and 1 and represent the relative intensity of a threat at any grid cell within the Spencer Gulf study area. For details on scaling method see glossary in Table 1 (main text). Maps were produced using R statistical software (version 3.3.1; https://www.r-project.org) and the packages raster[1], rgdal[2], sp[4] and rasterVis[6].

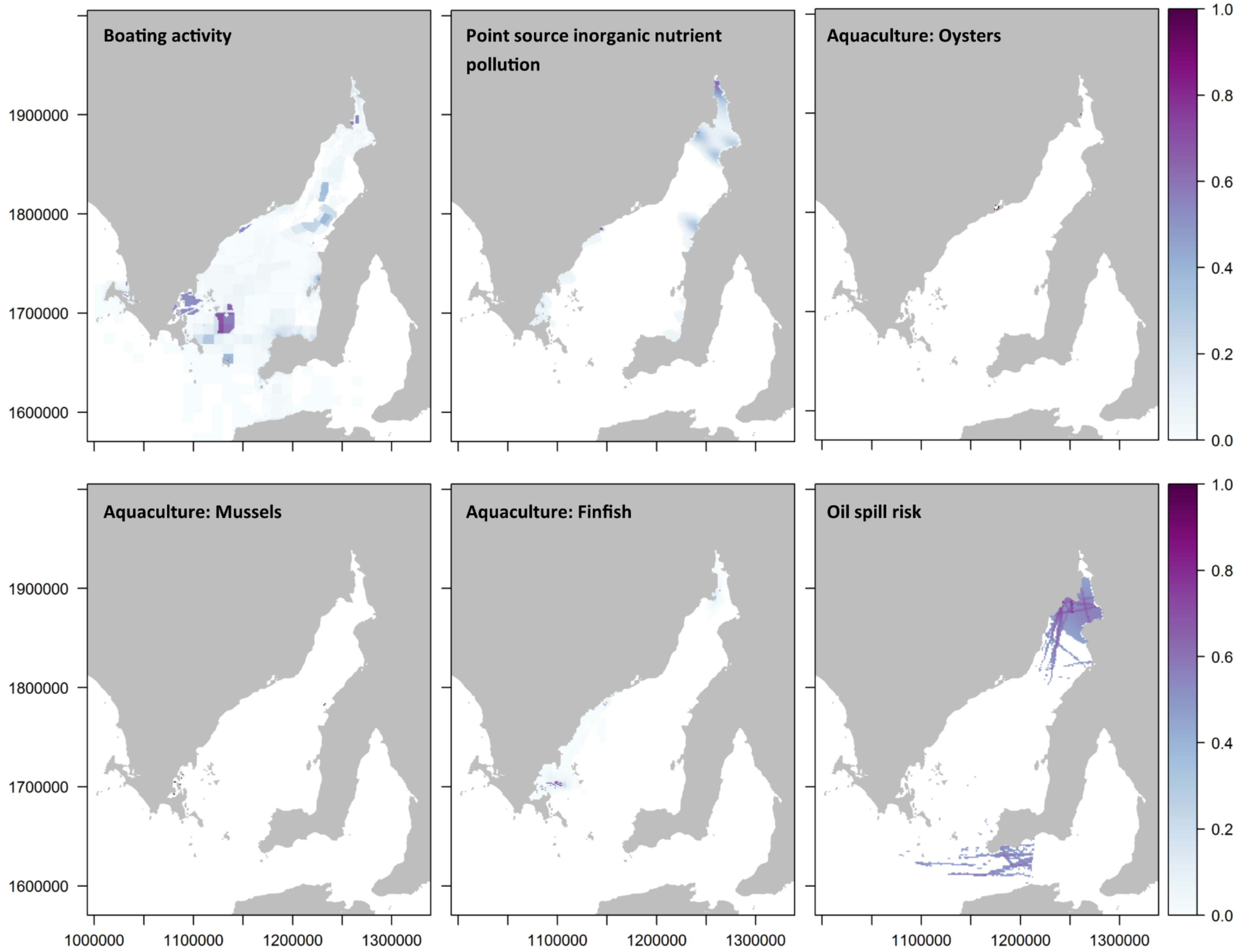

Supplementary Figure S3d: Threat spatial intensity maps continued. Values were scaled to between 0 and 1 and represent the relative intensity of a threat at any grid cell within the Spencer Gulf study area. For details on scaling method see glossary in Table 1 (main text). Maps were produced using R statistical software (version 3.3.1; https://www.r-project.org) and the packages raster[1], rgdal[2], sp[4] and rasterVis[6].

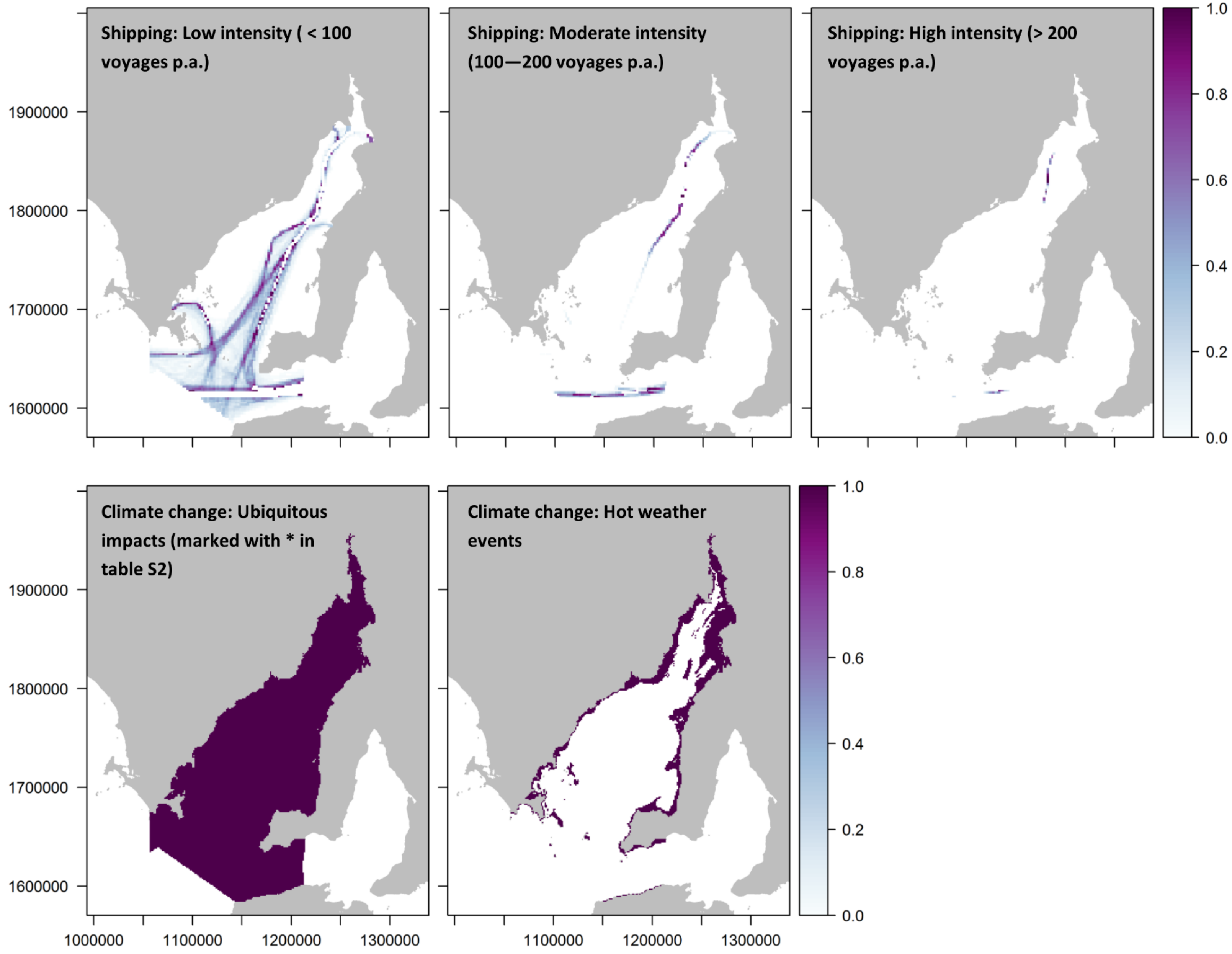

Supplementary Figure S3e: Threat spatial intensity maps continued. Values were scaled to between 0 and 1 and represent the relative intensity of a threat at any grid cell within the Spencer Gulf study area. For details on scaling method see glossary in Table 1 (main text). Maps were produced using R statistical software (version 3.3.1; https://www.r-project.org) and the packages raster[1], rgdal[2], sp[4] and rasterVis[6].

S2: Results

S2.1: Summary of the distribution of threats throughout Spencer Gulf

The exposure (presence/absence) and relative intensity of the various threat activities varied throughout the Gulf; therefore some areas (particularly the northern Gulf) were exposed to a greater number (Supplementary Figure S4a) and intensity (Supplementary Figure S4b) of threats than other areas.

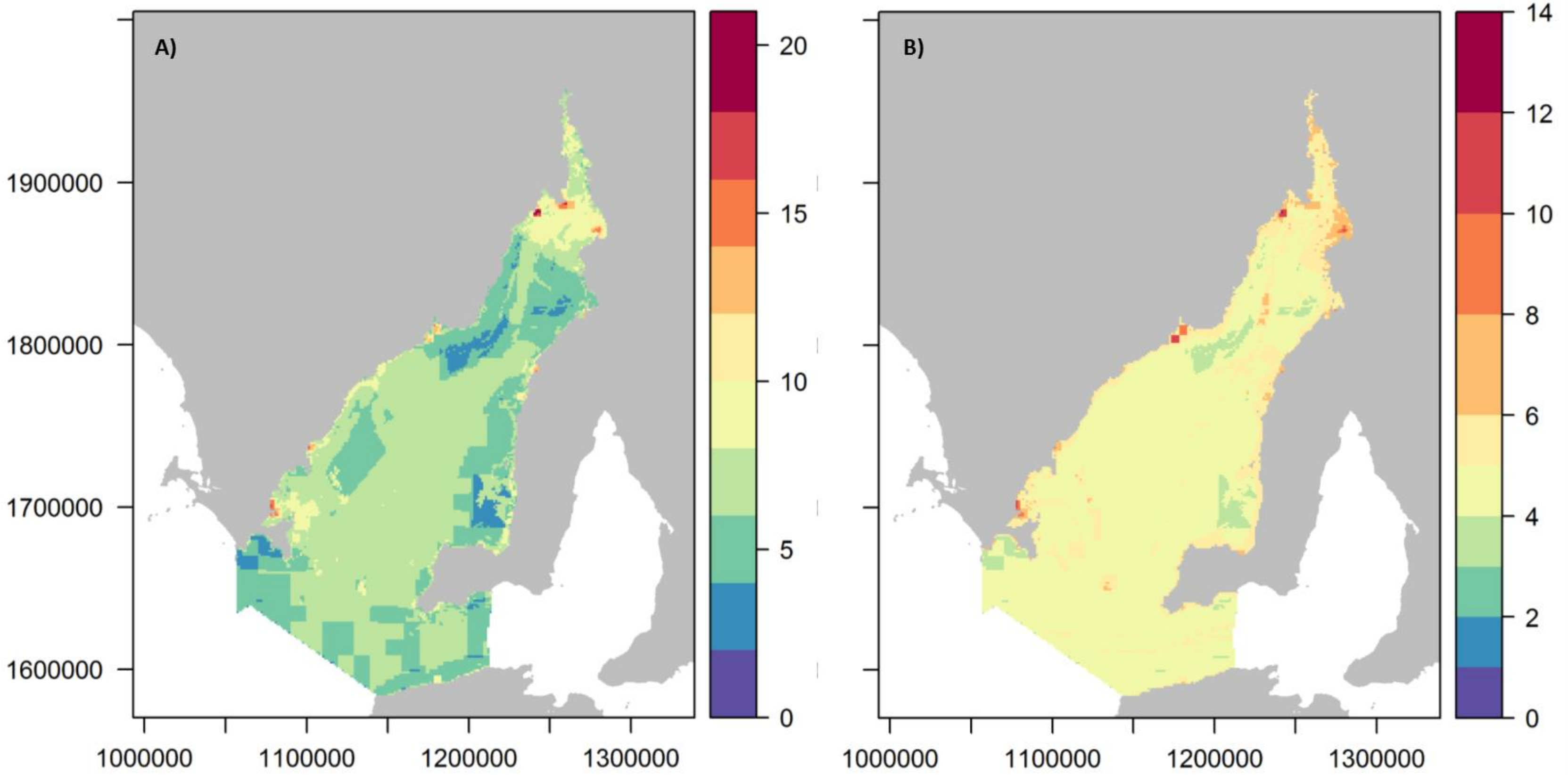

Supplementary Figure S4: Maps showing **A)** the total number of threats present in each grid cell and **B)** the summed threat intensity scores at each grid cell across the Spencer Gulf study area. Maps were produced using R statistical software (version 3.3.1; https://www.r-project.org) and the packages raster[1], rgdal[2], sp[4] and rasterVis[6].

S2.2: Sensitivity analyses

We tested the sensitivity of the 'most-likely' spatial cumulative impact assessment results to 1) the inclusion of expert-elicited 'effect' scores and 2) the inclusion/exclusion of each threat layer.

S2.2.1: Sensitivity of cumulative impact assessment results to the inclusion of expert-elicited effect scores.

We used a 'null model' approach where we gave all threats equal 'effect' scores, thereby removing the expert-elicited knowledge from the assessment and relying solely on the spatial exposure and intensity of the threats to infer cumulative impact. As would be expected, there were differences in the *absolute* values for cumulative impact scores when generated using the expert's effect scores and those generated using the equally-weighted (null) effect scores. However, when each cumulative impact map was scaled to be between 0 and 1 (using the min and max values), the relative spatial patterns of cumulative impact appeared visually similar throughout the Gulf for both benthic and pelagic ecosystems, regardless of whether expert-opinion or equal weighting methods were used. The resulting maps showed broadly the same areas of the Gulf as being at greatest impact from cumulative impacts in using both scoring methods (i.e. coastal ecosystems and areas of high shipping traffic or adjacent to industrial developments and population centres). Linear models supported this result, showing strong positive 'global' (i.e. all grid cells within the study area) correlation between the cumulative impact scores generated using the two different methods (expert scores vs null scores), for both benthic ($r = 0.834$, $p <0.001$) and pelagic ($r = 0.909$, $p <0.001$) ecosystems.

When we looked in more detail at the average cumulative impact scores from all cells within each ecosystem class, there were differences between the results produced when using the expert scores and those calculated using the equal effect score (null) method. Although the overall spatial patterns looked similar at a broad-scale (i.e. when looking at maps of the entire Gulf) and were strongly correlated when assessed at a global level (i.e. all grid cells within the study area), the ecosystem-

averaged scores and cumulative impact rankings changed notably when using the equal effect score (null) method. This can be seen by comparing Figure 4a in the main paper with Supplementary Figure S5 below.

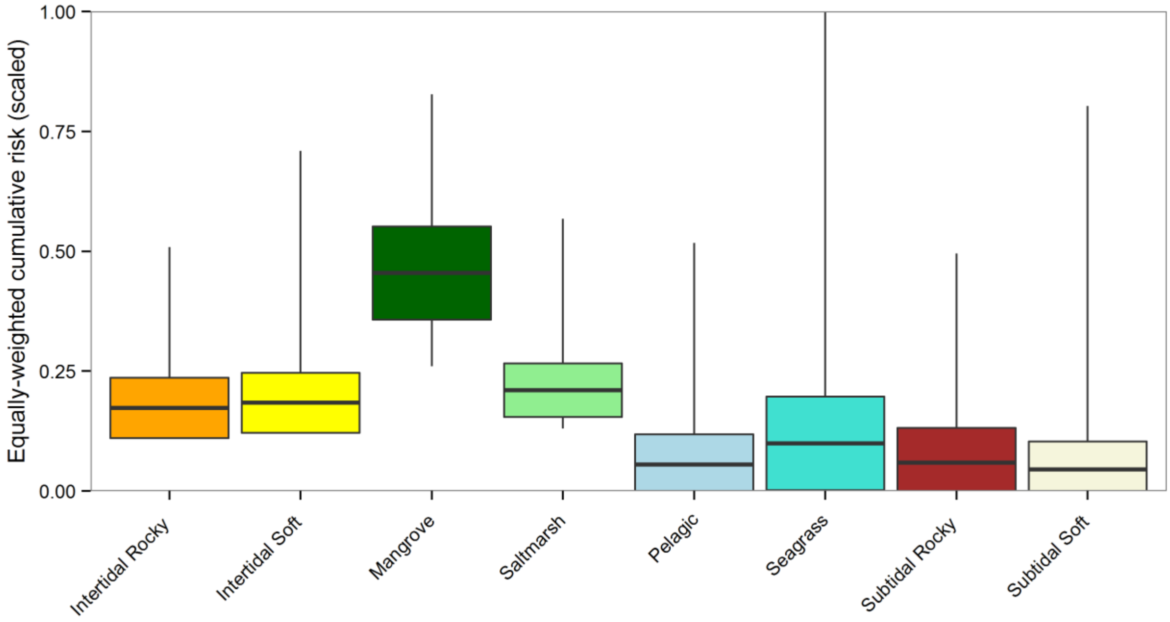

Supplementary Figure S5: Boxplot showing scaled cumulative impact scores for each ecosystem, based on equal-weighting of all threats to all ecosystems (i.e. the null approach). Data are summarised using the values of cumulative impact from all cells in each ecosystem class. The horizontal bold line is the mean value, box extent shows the standard deviation and the vertical lines extend to the min and max cumulative impact scores from any single cell in each ecosystem type.

When equal effect scores were used (i.e. no expert input), most ecosystems had similar averaged cumulative impact scores, except mangrove, which was noticeably higher (Supplementary Figure S5). This contrasts with the cumulative impact scores calculated using the expert-elicited effect scores, where there was greater differentiation in averaged cumulative impact between the ecosystems, and the ecosystem that was deemed to be most impacted was intertidal rocky (Supplementary Figure 4 main paper). This outcome indicates that the assessment results were sensitive to the inclusion of expert-elicited effect scores. Without empirical data to ground-truth the results, there is no way to be sure which of the scoring methods (expert scores vs null scores) is most appropriate. However, it seems reasonable to expect that different ecosystems would respond differently to different threats[11,12] and thus it appears most likely that the inclusion of expert-elicited effect scores will achieve

more realistic results. Without the expert's effect scores, the nuances of the impact from different threats to different ecosystems are lost from the analysis.

S2.2.2: Sensitivity of cumulative impact assessment results to the threat layers included in the analysis.

To test for the relative influence of each of the threat layers, we used a jack-knife technique, where each threat was removed from the assessment in turn, and the cumulative impact was re-calculated, leaving out the scores attributable to the removed threat.

We compared the global correlation of the cumulative impact maps created for each jack-knife iteration with the original (most-likely) cumulative impact map, which included all threat layers. The results of these correlations are given in the last column of Supplementary Table S3 (as R^2 values). The majority of jack-knife test outputs were very strongly correlated with the results from the assessment that included all threat layers; with 89 % of the jack-knife results having a correlation of ≥ 0.95, for both the benthic and pelagic cumulative impact scores (Supplementary Table S3). Orange text in Supplementary Table S3 highlights the top 5 most influential threat layers. These are the threats which, when removed from the analysis, resulted in the lowest correlation coefficients with the original (most-likely) assessment results.

Supplementary Table S3: Contribution of each threat to the most-likely cumulative impact score across all pixels classified as either 1) benthic or 2) pelagic ecosystems. Footprint = the total area of Spencer Gulf exposed to the threat at any intensity level. Images showing the exposure and intensity of each threat layer are provided in Supplementary Figures S3a –e. R^2 values were calculated during jack-knife sensitivity testing (see supplementary methods section S2.2.2) and indicate the influence of each threat layer on the final ('most-likely' scenario) cumulative impact scores. In this context, lower R^2 values indicate a larger influence (i.e. a greater difference after the threat was removed from the assessment). Orange text highlights the top five threats to the benthic and pelagic ecosystems.

	**Footprint (km^2)**	**Min**	**Max**	**Mean**	**Median**	**SD**	**Sum**	**R^2**
**Threat layer**	**Benthic ecosystems (combined) scores**							
Climate change: hot weather events	5190.3	< 0.1	4.25	0.73	0.00	1.46	271264	0.552
Climate change: extreme rainfall events	30361.2	0.00	2.88	0.42	0.00	0.83	157525	0.743

	Footprint (km²)	Min	Max	Mean	Median	SD	Sum	R^2
**Threat layer**	**Benthic ecosystems (combined) scores**							
Pollution: oil	3175.4	0.00	4.17	0.22	0.00	0.67	81049	0.893
Pollution: nutrients	3614.4	0.00	4.73	0.07	0.00	0.25	26843	0.957
Climate change: increased average temp	30361.2	1.83	4.00	3.05	2.85	0.31	1133269	0.966
Introduced marine species: fouling species	235.6	0.00	3.55	0.03	0.00	0.22	11692	0.976
Climate change: decreased average rainfall	29767.6	< 0.1	2.50	0.06	0.00	0.36	20927	0.978
Fishing: bottom trawl (prawn)	12113.3	0.00	4.77	0.15	0.00	0.53	57200	0.981
Marine habitat modification: jetties	267.8	0.00	5.25	0.02	0.00	0.19	6384	0.983
Acid sulphate soil disturbance	516.0	0.00	3.75	0.03	0.00	0.22	10931	0.984
Coastal activities	1428.9	0.00	3.75	0.02	0.00	0.16	7747	0.986
Introduced marine species: filter feeders	296.0	0.00	3.13	0.01	0.00	0.14	5057	0.989
Pollution: metals	1877.3	0.00	2.85	0.01	0.00	0.08	4639	0.990
Coastal habitat modification	345.9	0.00	5.38	0.01	0.00	0.19	3490	0.991
Introduced marine species: predators & parasites	172.0	0.00	1.92	0.01	0.00	0.10	2797	0.991
Boating activity	30361.2	0.00	2.96	0.10	0.03	0.22	37886	0.992
Shipping: moderate intensity	1430.8	0.00	2.90	0.02	0.00	0.21	8982	0.995
Shipping: high intensity	160.7	0.00	3.54	0.01	0.00	0.15	3099	0.996
Marine habitat modification: marinas	140.9	0.00	4.50	0.00	0.00	0.07	1328	0.996
Marine habitat modification: ports and harbours	365.5	0.00	4.47	0.00	0.00	0.06	1466	0.997
Fishing: lobster pots	950.2	0.00	2.00	0.01	0.00	0.07	2310	0.998
Finfish aquaculture	2597.3	0.00	4.53	0.01	0.00	0.10	4372	>0.999
Oyster aquaculture	7.3	0.00	3.75	0.00	0.00	0.05	328	>0.999
Climate change: ocean acidification	30361.2	< 0.1	4.00	2.83	3.08	0.49	1050105	>0.999
Fishing: hand collection (abalone)	960.5	0.00	1.64	0.00	0.00	0.01	217	>0.999
Pollution: thermal	2.5	0.00	3.13	0.00	0.00	0.02	78	>0.999

	Footprint (km^2)	Min	Max	Mean	Median	SD	Sum	R^2
**Threat layer**	**Benthic ecosystems (combined) scores**							
Shipping: low intensity	13094.2	0.00	2.11	0.14	0.00	0.29	50822	>0.999
**Threat layer**	**Pelagic ecosystem scores**							
Climate change: ocean acidification	30361.2	3.75	3.75	3.75	3.75	0.00	1666710	0.669
Climate change: increased average temperature	30361.2	3.33	3.33	3.33	3.33	0.00	1481372	0.706
Climate change: extreme rainfall events	30361.2	2.75	2.75	2.75	2.75	0.00	1222254	0.757
Climate change: hot weather events	5190.3	0.00	3.42	0.41	0.00	1.11	180800	0.951
Pollution: oil	3175.4	0.00	3.84	0.24	0.00	0.73	107722	0.971
Shipping: low intensity	13094.2	0.00	1.25	0.14	0.00	0.25	62895	0.988
Boating activity	30361.2	0.00	2.36	0.11	0.03	0.26	48175	0.989
Fishing: bottom trawl (prawn)	12113.3	0.00	3.58	0.10	0.00	0.39	45474	0.989
Fishing: seine net (sardine)	16870.7	0.00	2.42	0.07	0.00	0.23	32519	0.993
Pollution: nutrients	3614.4	0.00	2.61	0.04	0.00	0.15	16519	0.995
Shipping: moderate intensity	1430.8	0.00	2.42	0.04	0.00	0.24	18939	0.996
Shipping: high intensity	160.7	0.00	3.67	0.01	0.00	0.16	4729	0.999
Finfish aquaculture	2597.3	0.00	3.50	0.01	0.00	0.09	4125	0.999
Pollution: metals	1877.3	0.00	3.00	0.01	0.00	0.06	2978	0.999
Marine habitat modification: jetties	267.8	0.00	2.42	0.00	0.00	0.08	2090	0.999
Marine habitat modification: ports and harbours	365.5	0.00	3.58	0.00	0.00	0.05	1040	1.000
Marine habitat modification: marinas	140.9	0.00	2.83	0.00	0.00	0.03	455	1.000
Mussel aquaculture	14.9	0.00	2.17	0.00	0.00	0.05	490	1.000

S2.3: Detailed maps of cumulative impact around industrial and population centres

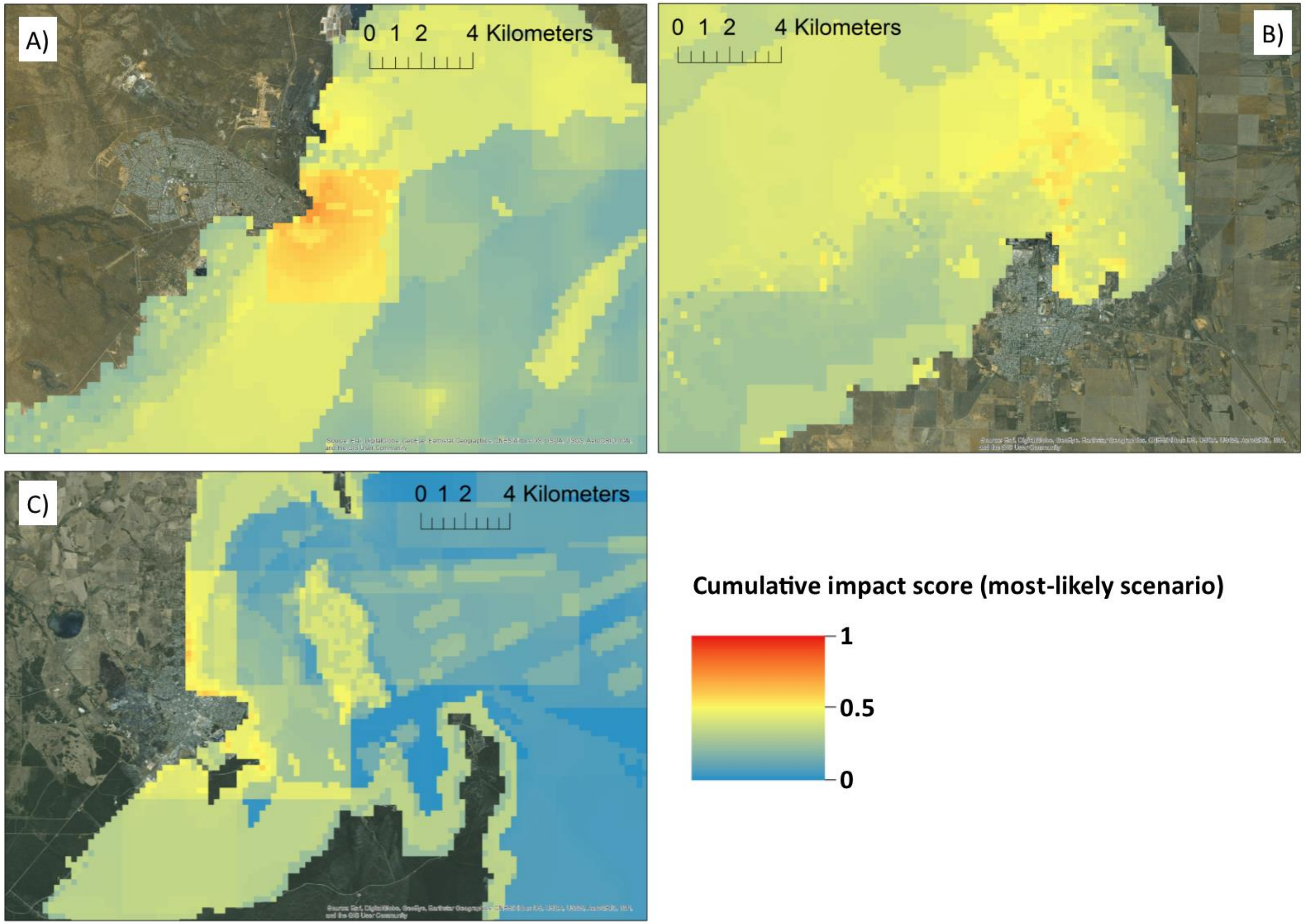

Supplementary Figure S6: Zoomed-in maps of most-likely cumulative impacts on benthic ecosystems. The mapped areas correspond to the boxed locations in Figure 3b of the main text and were selected to highlight the greater cumulative impact scores close to the population and industrial centres in A) Whyalla, B) Port Pirie and C) Port Lincoln. Background map source/credit: ESRI, DigitalGlobe, Earthstar Geographics, CNES/Airbus DS, USDA, AeroGRID, IGN and the GIS user community. Maps were created using ArcGIS® software by Esri[13] (www.esri.com). ArcGIS® and ArcMap™ are the intellectual property of Esri and are used herein under license. Copyright © Esri. All rights reserved.

S2.4: Testing for symmetry in experts' uncertainty around most-likely scores

To assess whether the experts' best- and worst-case scores were symmetrical around the most likely scores we calculated and compared 'difference values'. For each threat and ecosystem pair, 'lower difference' was calculated as the difference between the most-likely score and the best-case score, and 'upper difference' was calculated as the difference between the worst-case score and the most-likely score. We plotted these difference values against each other, expecting a linear relationship if the best-case and worst-case scores were equally different from the most-likely score (i.e. symmetrical). In fact, what we found was that there was no clear relationship between the upper and lower differences, indicating that the most likely score was often not equidistant from the best-case and worst-case scores (Supplementary Figure S7). This asymmetry was not affected by the value of the most-likely score (Supplementary Figure S7).

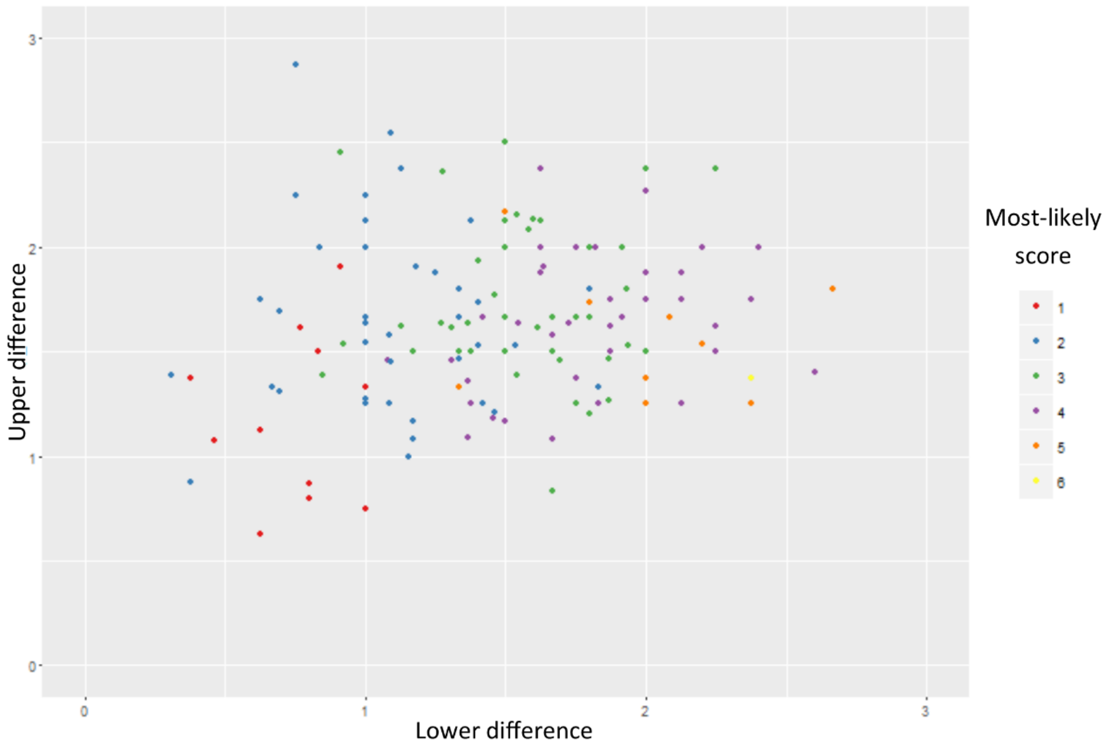

Supplementary Figure S7: Scatter plot of the 'upper difference' value (difference between the experts' average worst-case score and average most-likely score for a given threat and ecosystem pair) against the 'lower difference' value (difference between the experts' average most-likely score and average best-case score for a given threat and ecosystem pair). Points are coloured by the average most-likely score (rounded to the nearest integer).

S2.5: Beta distributed uncertainty simulations

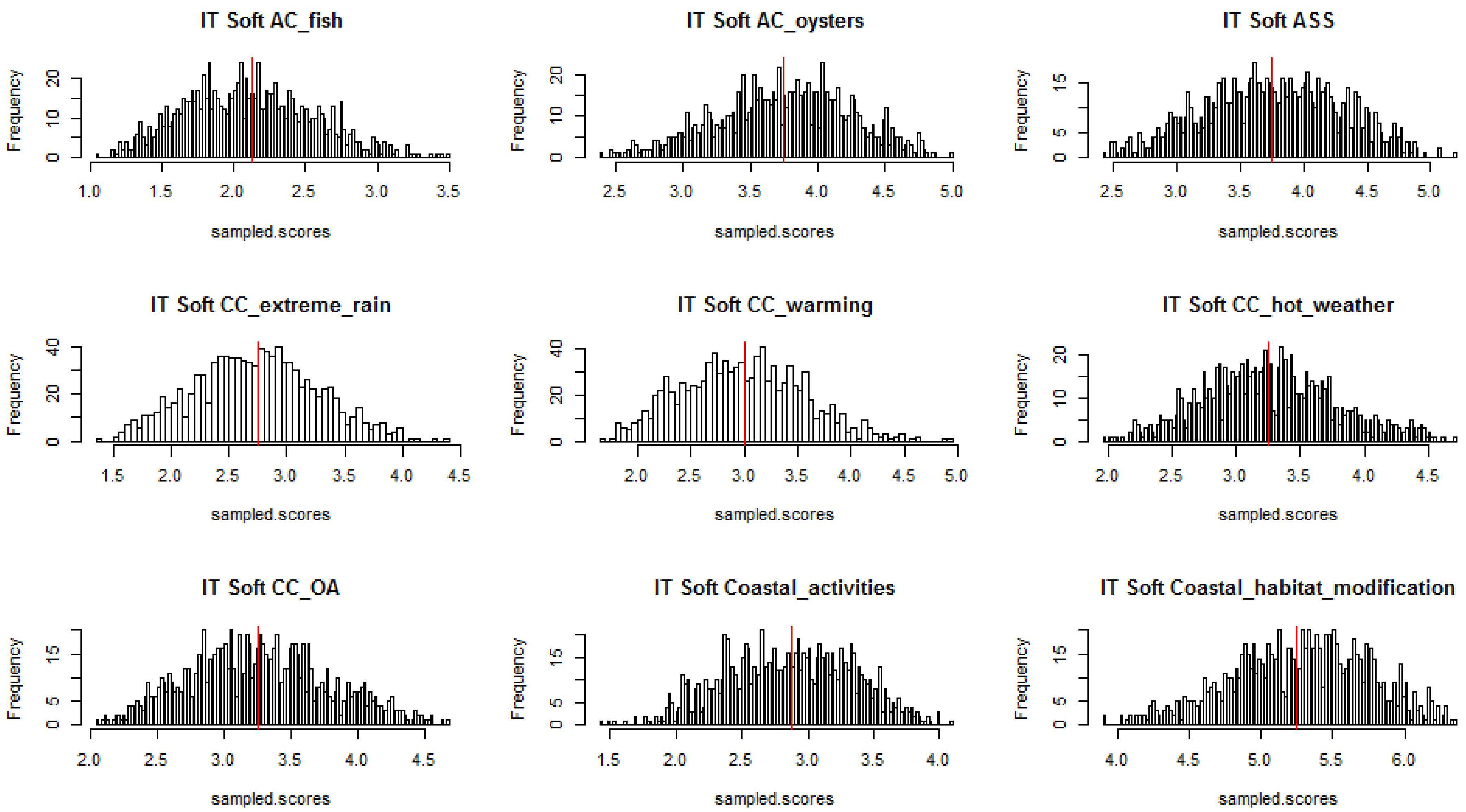

Supplementary Figure S8: Histograms of N = 1000 effect scores sampled from beta distributions parameterised to be bounded by the best-case (lower) and worst-case (upper) expert effect scores and with highest frequency density around the most-likely scores (indicated by the red vertical line). Examples shown are for the intertidal soft ('IT Soft') ecosystem and nine randomly selected threats: AC_fish = finfish aquaculture; AC_oysters = oyster aquaculture; ASS = acid sulphate soil; CC_extreme_rain = Climate change – extreme rainfall events; CC_warming = Climate change – global warming; CC_hot_weather = Climate change – hot weather events; CC_OA = Climate change – ocean acidification; other names are self-explanatory.

References

1. Hijmans, R. J. raster: Geographic Data Analysis and Modeling, v.2.5-8. (2016).
2. Bivand, R., Keitt, T. & Rowlingson, B. rgdal: Bindings for the Geospatial Data Abstraction Library. R package version 0.8-16. (2014).
3. Gillanders, B. M. *et al.* Spencer Gulf Research Initiative: Development of an Ecosystem Model for Fisheries and Aquaculture. (Fisheries Research and Development Corporation. , 2015).
4. Bivand, R. S., Pebesma, E. & Gomez-Rubio, V. *Applied spatial data analysis with R* (Springer, 2013).
5. O'Connell, L. G. *et al.* Oceanographic controls on shallow-water temperate carbonate sedimentation: Spencer Gulf, South Australia. *Sedimentology* **63**, 105-135 (2016).
6. Perpinan L.O. & Hijmans, R. rasterVis: Visualization methods for the raster package. R package version 0.28. (2014).
7. Wiltshire, K. H., Rowling, K. P. & Deveney, M. R. Introduced marine species in South Australia: a review of records and distribution mapping. (South Australian Research and Development Institute (Aquatic Sciences), Adelaide, South Australia, 2010).
8. Halpern, B. S. *et al.* A global map of human impact on marine ecosystems. *Science* **319**, 948-952 (2008).
9. Gaylard, S. in *Natural History of Spencer Gulf* (eds S. A. Shepherd *et al.*) (Royal Society of South Australia Inc., 2015).
10. Middleton, J., Doubell, M., James, C., Luick, J. & van Ruth, P. PIRSA Initiative II: carrying capacity of Spencer Gulf: hydrodynamic and biogeochemical measurement modelling and performance monitoring. Report No. SARDI Research Report Series No. 705, (SARDI Aquatic Sciences, SA, 2013).
11. Halpern, B. S., Selkoe, K. A., Micheli, F. & Kappel, C. V. Evaluating and ranking the vulnerability of global marine ecosystems to anthropogenic threats. *Conserv Biol* **21**, 1301-1315 (2007).
12. Teck, S. J. *et al.* Using expert judgment to estimate marine ecosystem vulnerability in the California Current. *Ecological Applications* **20**, 1402-1416 (2010).
13. ArcGIS Desktop: Release 10.3.1. (Environmental Systems Research Institute, Redlands, CA, 2014).
